# Supplementary material for: Sesquiterpenoids and Their Anti-Inflammatory Activity: Evaluation of Ainsliaea yunnanensis
Source: Molecules. 2019 May 1;24(9):1701. doi: 10.3390/molecules24091701 (PMC6539984; doi:10.3390/molecules24091701)
Supplement: Supplementary file 1 [file molecules-24-01701-s001.zip › molecules-489408-proofreading done-Suppl/data of compounds 1-10/chromatograms of compounds 2-4.pdf]

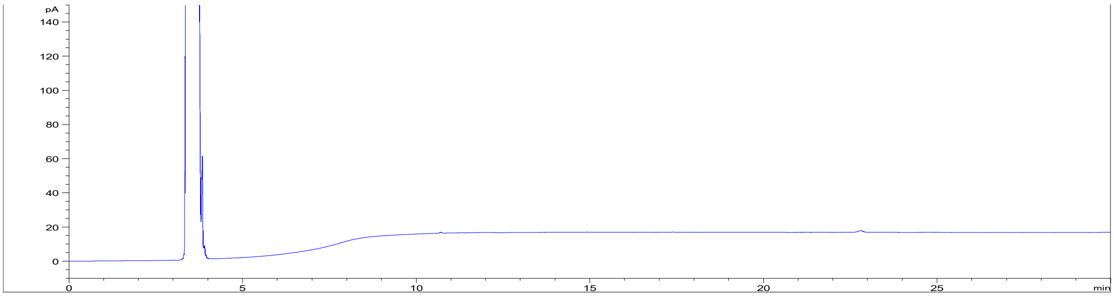

Figure 1: Chromatogram of blank solvent

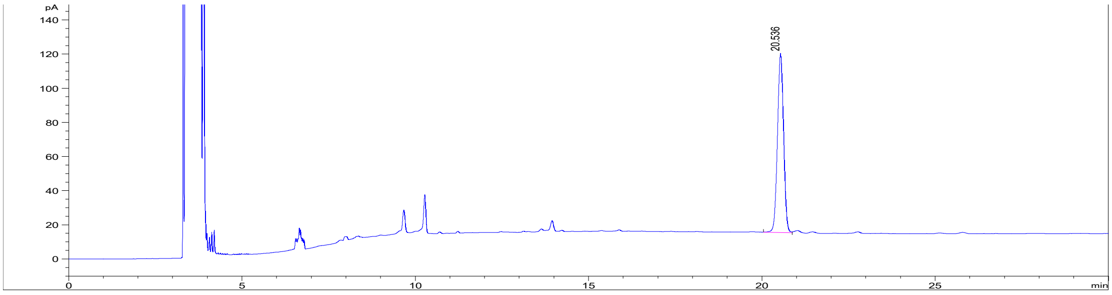

Figure 2: Chromatogram of D-glucose

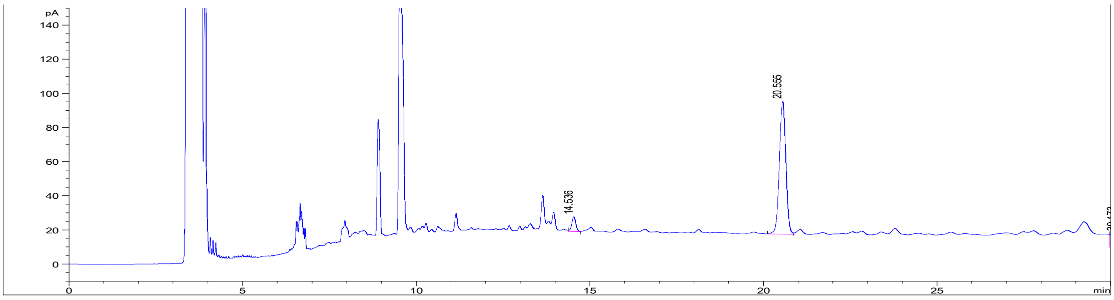

Figure 3: Chromatogram of Compound 1

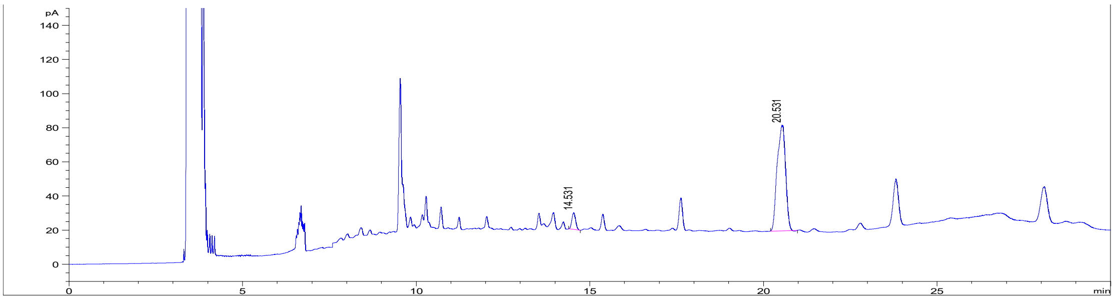

Figure 4: Chromatogram of Compound 2

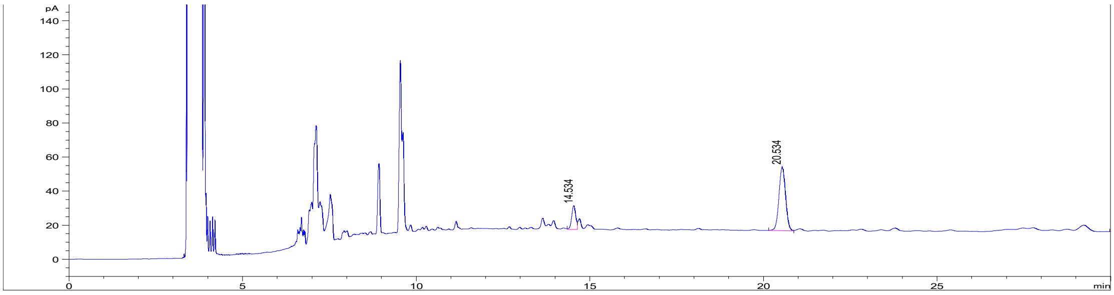

Figure 5: Chromatogram of Compound 3
