# Supplementary material for: Sesquiterpenoids and Their Anti-Inflammatory Activity: Evaluation of Ainsliaea yunnanensis
Source: Molecules. 2019 May 1;24(9):1701. doi: 10.3390/molecules24091701 (PMC6539984; doi:10.3390/molecules24091701)
Supplement: Supplementary file 1 [file molecules-24-01701-s001.zip › molecules-489408-proofreading done-Suppl/data of compounds 1-10/Compound 4-NOE.pdf]

ROESYPHSW DMSO D:\\ shangxiaoya 38

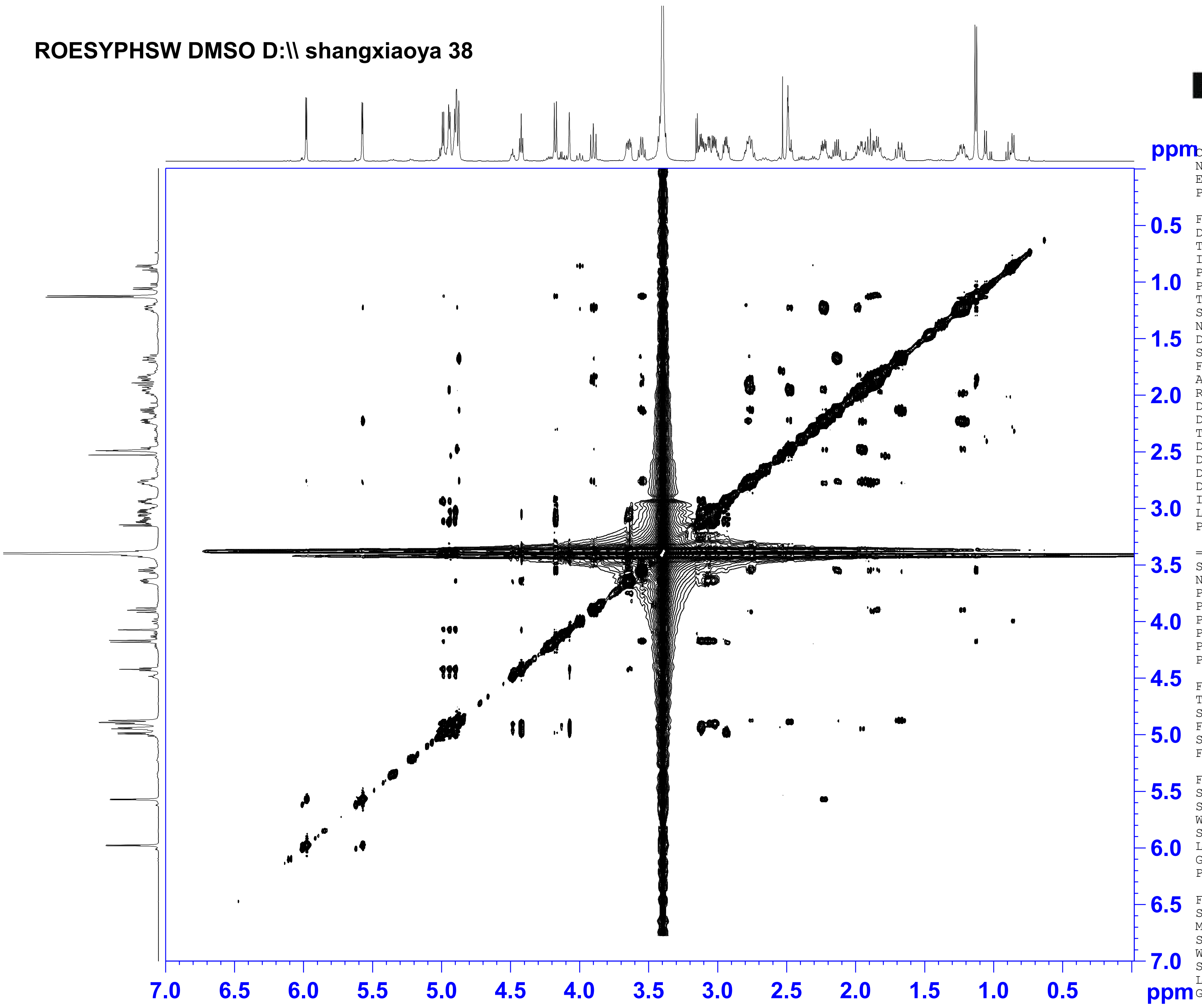


```
Current Data Parameters
NAME        20190128-TRF-26
EXPNO                     2
PROCNO                    1

F2 - Acquisition Parameters
Date_              20190130
Time                   6.02
INSTRUM               spect
PROBHD      5 mm CPPTCI 1H
PULPROG         roesyphpp.2
TD                     2048
SOLVENT                DMSO
NS                       32
DS                       32
SWH              3501.401 Hz
FIDRES           1.709668 Hz
AQ              0.2924544 sec
RG                       64
DW                  142.800 usec
DE                    10.00 usec
TE                    298.2 K
D0               0.00013371 sec
D1               1.00000000 sec
D11              0.03000000 sec
D12              0.00002000 sec
IN0              0.00028560 sec
L4                     1000
P15               200000.00 usec

======== CHANNEL f1 ========
SFO1            500.0616502 MHz
NUC1                     1H
P1                     8.00 usec
P17                 2500.00 usec
P25                  100.00 usec
PLW1             8.69999981 W
PLW10            0.61866999 W
PLW27            0.22272000 W

F1 - Acquisition parameters
TD                      256
SFO1               500.0617 MHz
FIDRES            13.677346 Hz
SW                    7.002 ppm
FnMODE           States-TPPI

F2 - Processing parameters
SI                     1024
SF              500.0600091 MHz
WDW                   QSINE
SSB                       2
LB             0 Hz
GB             0
PC                     1.00

F1 - Processing parameters
SI                     1024
MC2             States-TPPI
SF              500.0600091 MHz
WDW                   QSINE
SSB                       2
LB             0 Hz
GB             0
```
