# Supplementary figures and images for: Sesquiterpenoids and Their Anti-Inflammatory Activity: Evaluation of Ainsliaea yunnanensis
Source: Molecules. 2019 May 1;24(9):1701. doi: 10.3390/molecules24091701 (PMC6539984; doi:10.3390/molecules24091701)

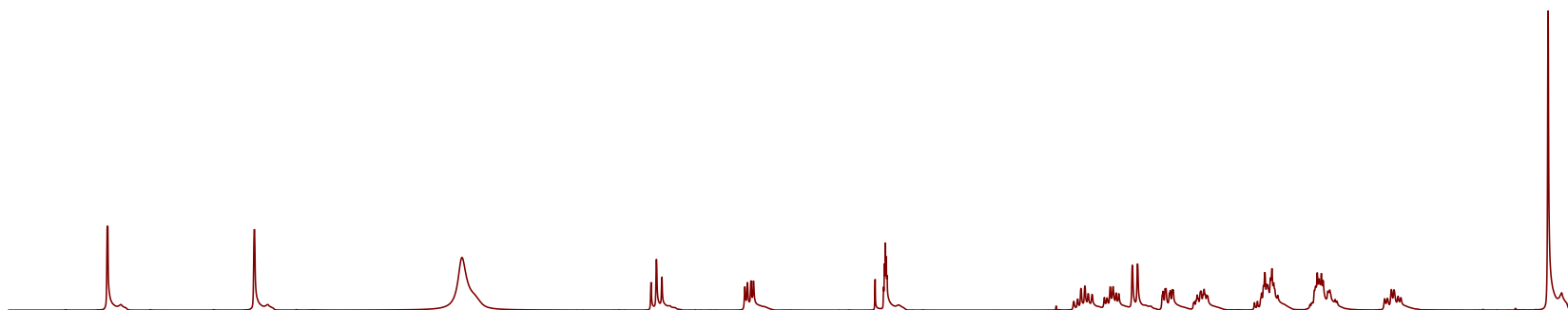

20171101-TRF-29  
BRUKER AV-III-500 COSY-NMR TRF-29 IN DMSO 2017.11.01  
HMBCGPND DMSO D:\\shangxiaoya 12

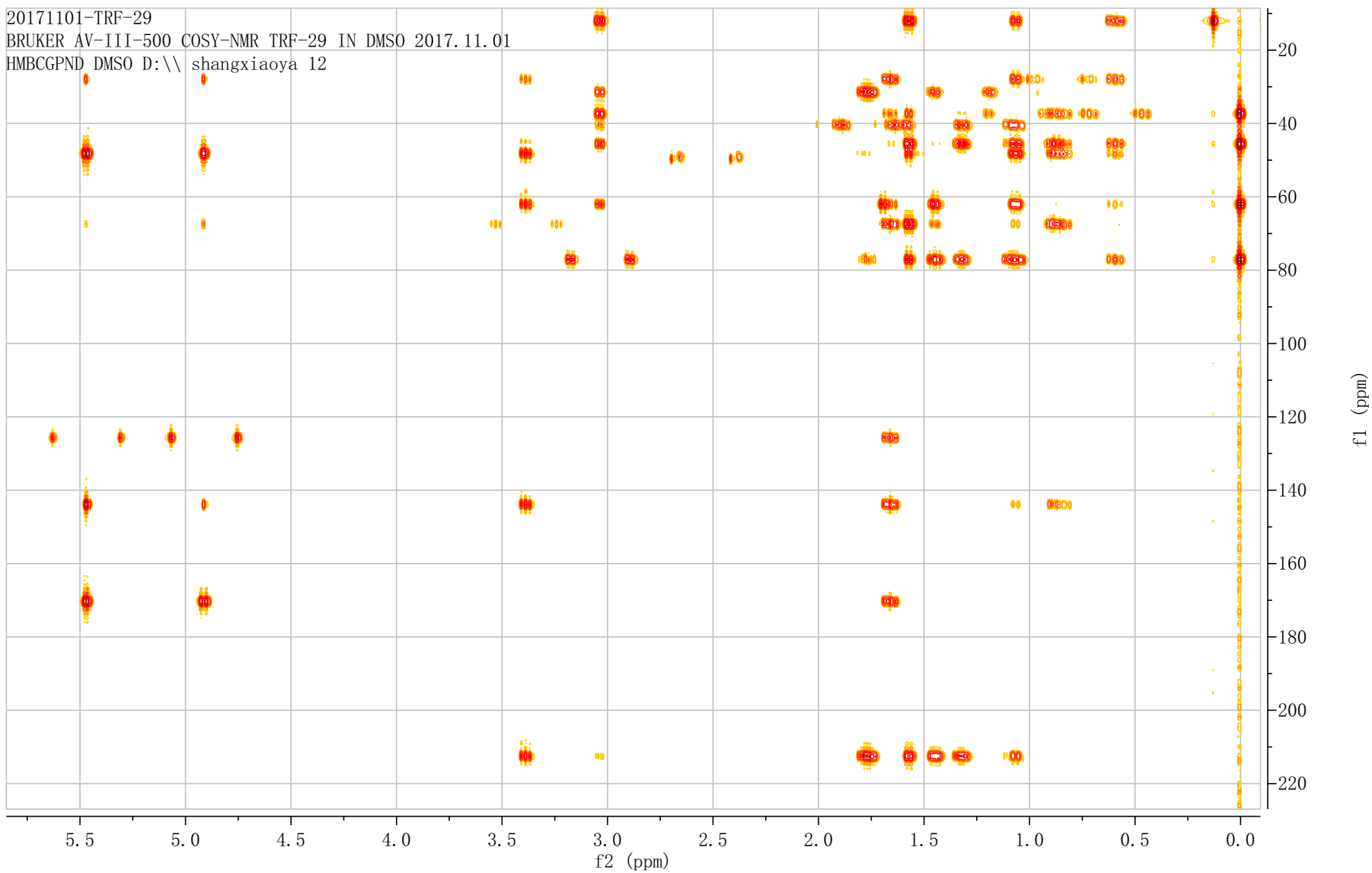

Supplement: Supplementary file 1 [file molecules-24-01701-s001.zip › molecules-489408-proofreading done-Suppl/data of compounds 1-10/Compound 1-BC.pdf]

20170908-TRF-29  
BRUKER AV-III-500 13C-NMR TRF-29 IN MeOD 2017.09.08  
13C-NMR MeOD D:\\ shangxiaoya 21

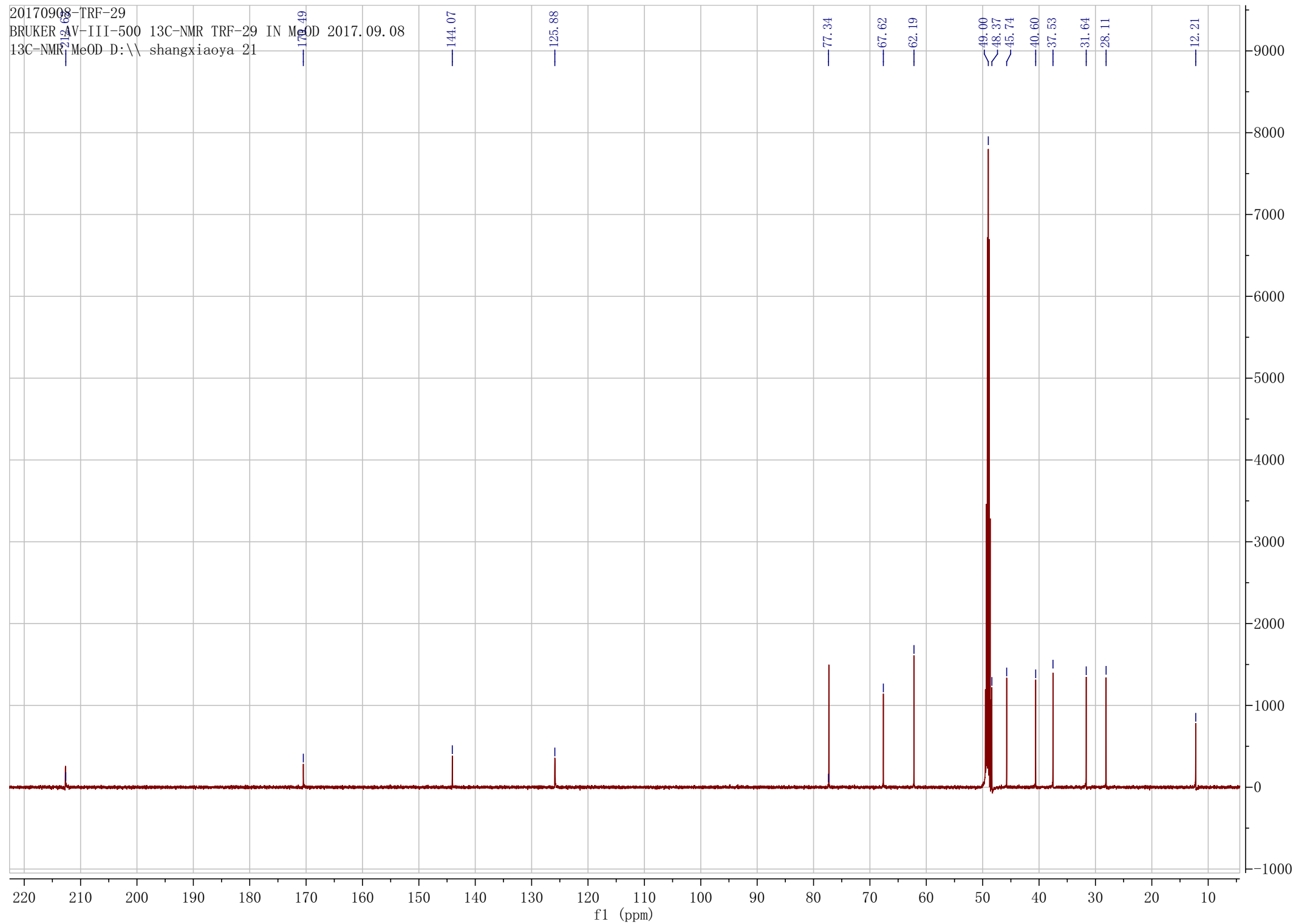

Supplement: Supplementary file 1 [file molecules-24-01701-s001.zip › molecules-489408-proofreading done-Suppl/data of compounds 1-10/Compound 1-C.pdf]

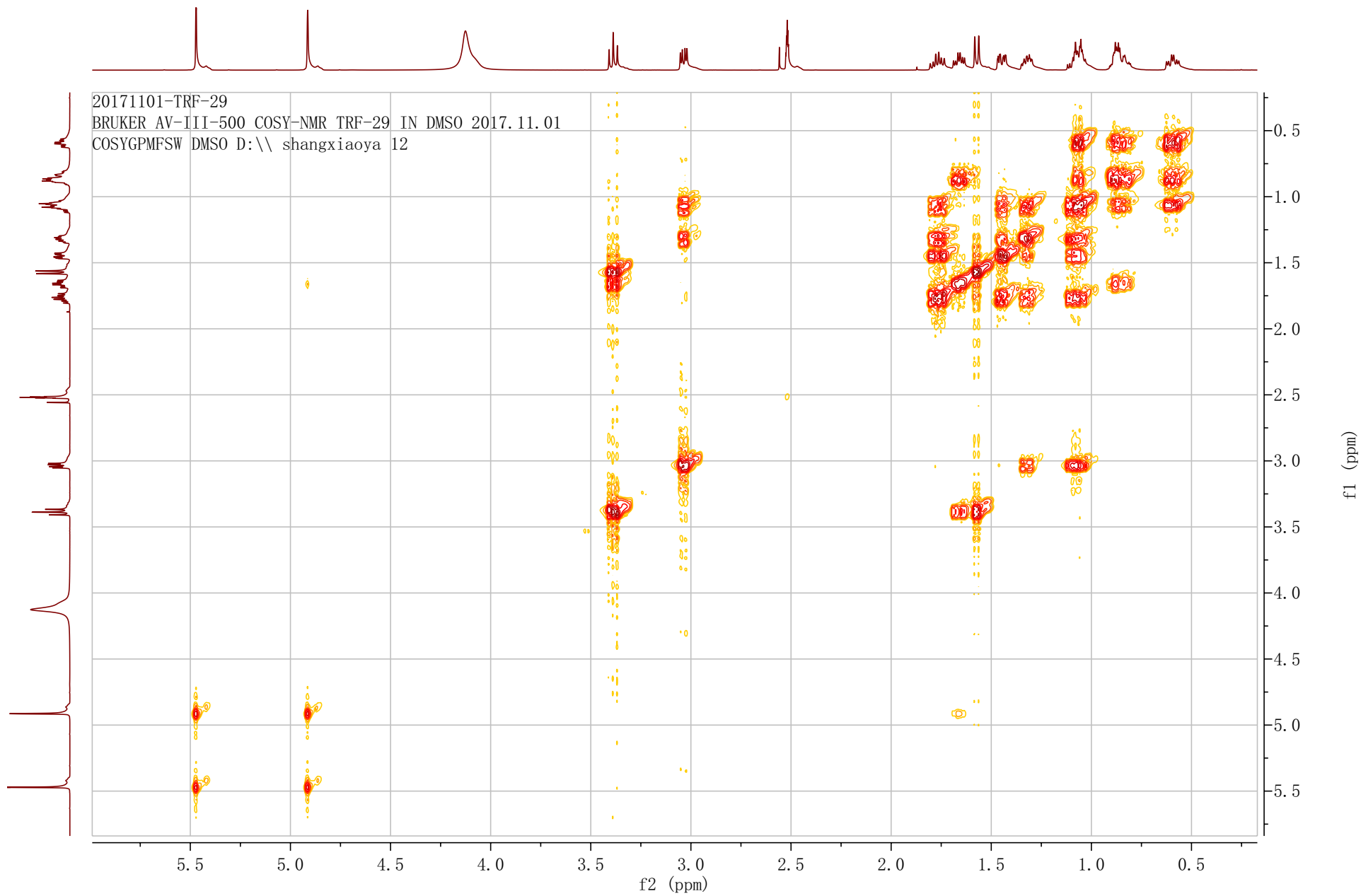

Supplement: Supplementary file 1 [file molecules-24-01701-s001.zip › molecules-489408-proofreading done-Suppl/data of compounds 1-10/Compound 1-COSY.pdf]

20170908+TRF-29  
BRUKER AV-III-500 1H-NMR TRF-29 IN MeOD 2017.09.08  
1H-NMR MeOD D:\\\\shangxiaoya 21

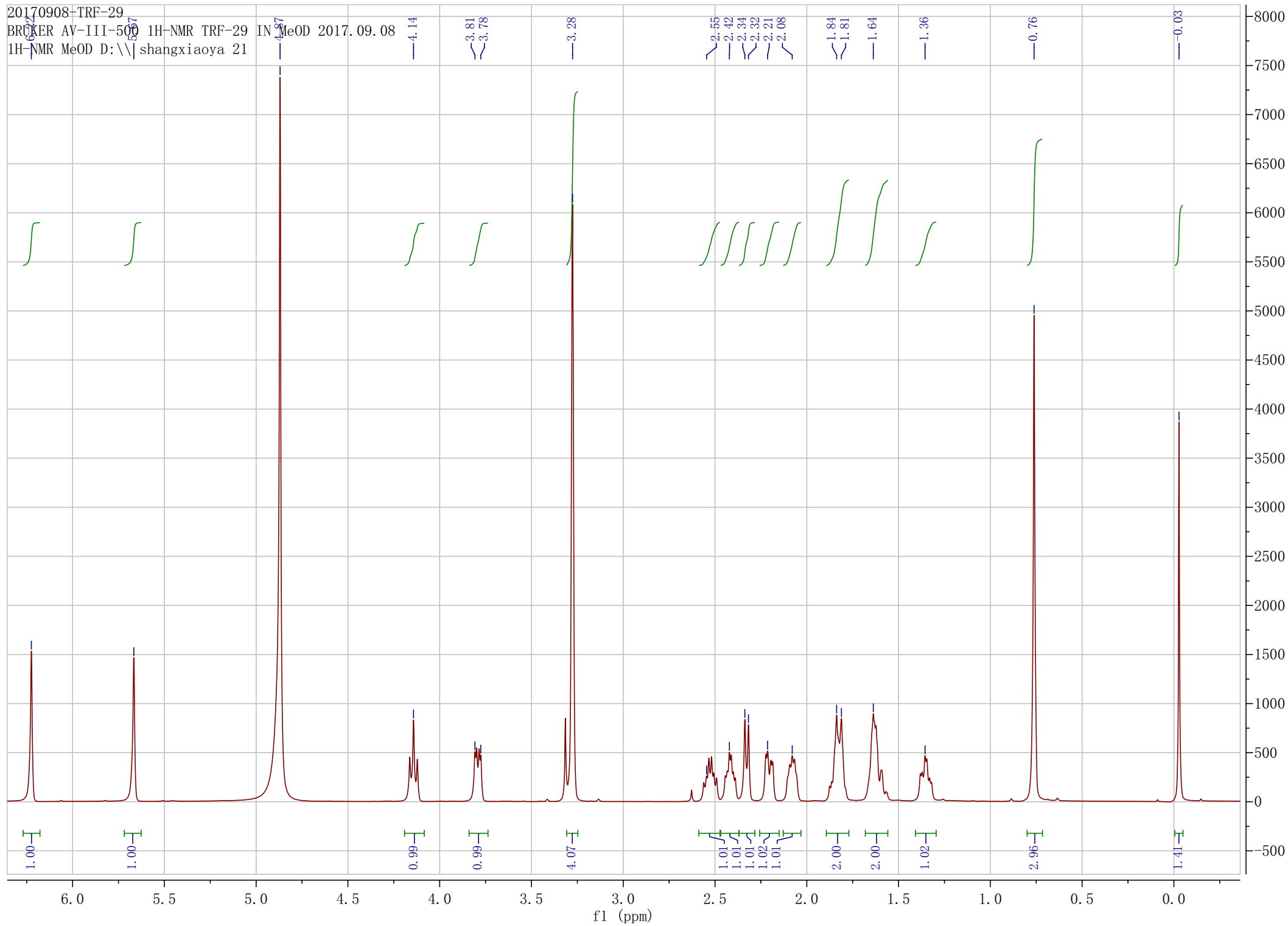

Supplement: Supplementary file 1 [file molecules-24-01701-s001.zip › molecules-489408-proofreading done-Suppl/data of compounds 1-10/Compound 1-H.pdf]

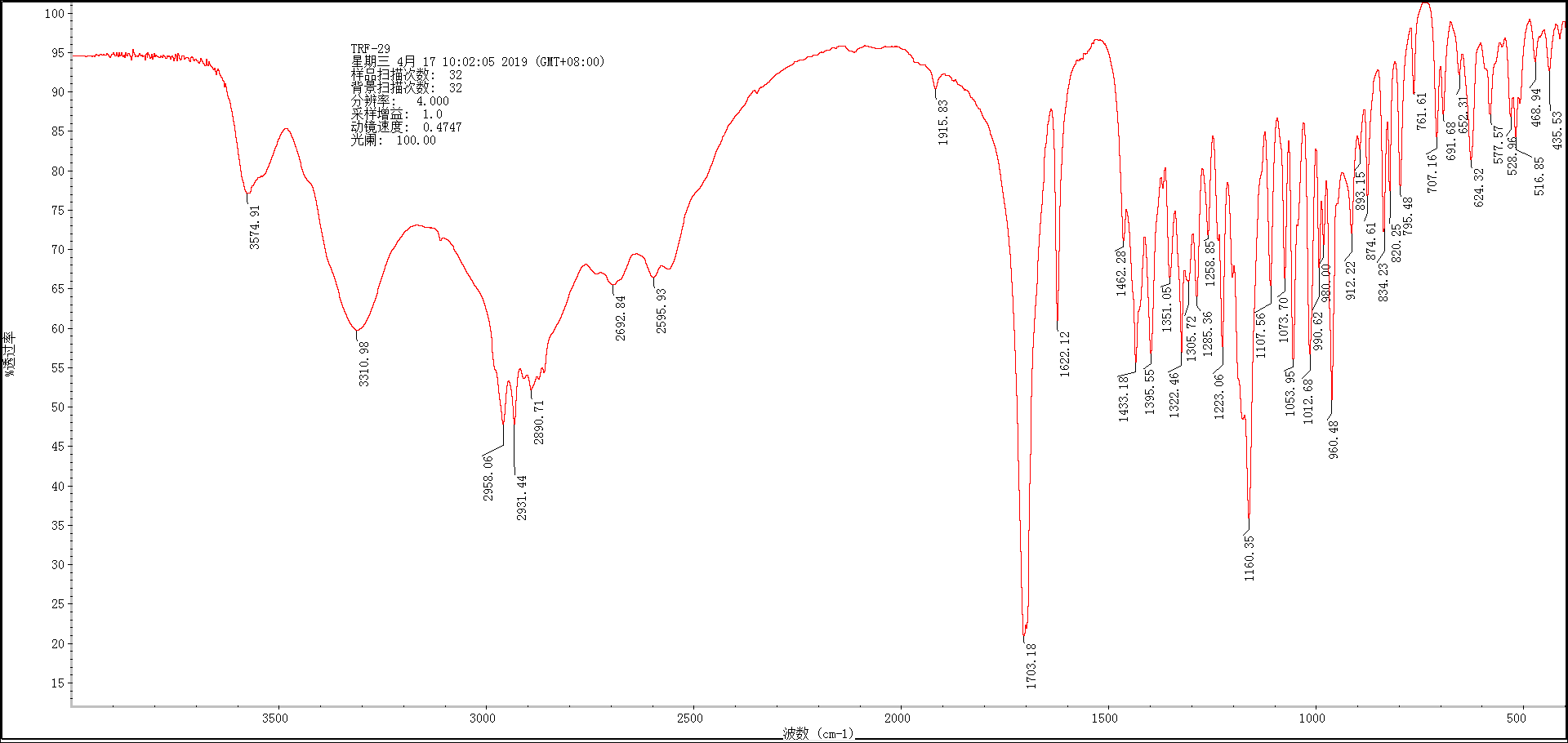

Supplement: Supplementary file 1 [file molecules-24-01701-s001.zip › molecules-489408-proofreading done-Suppl/data of compounds 1-10/Compound 1-IR.TIF]

TRF-29\_Pos\_FullMs #437 RT: 2.21 AV: 1 NL: 1.09E9  
T: FTMS + p ESI Full ms [50.0000-500.0000]

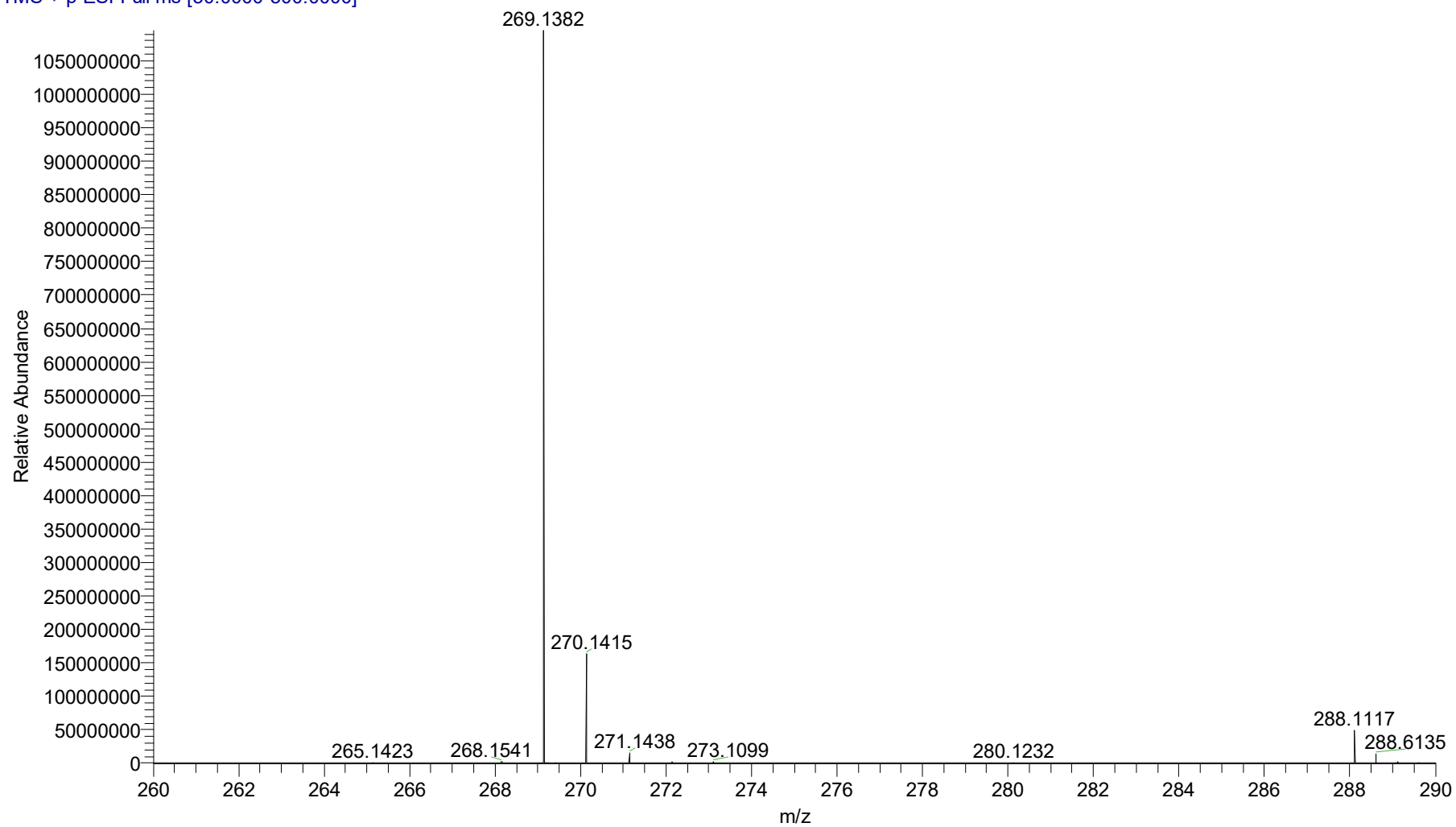

Supplement: Supplementary file 1 [file molecules-24-01701-s001.zip › molecules-489408-proofreading done-Suppl/data of compounds 1-10/Compound 1-MS.pdf]

20190128-TRF-29

ROESYPHSW MeOD D:\\ shangxiaoya 37

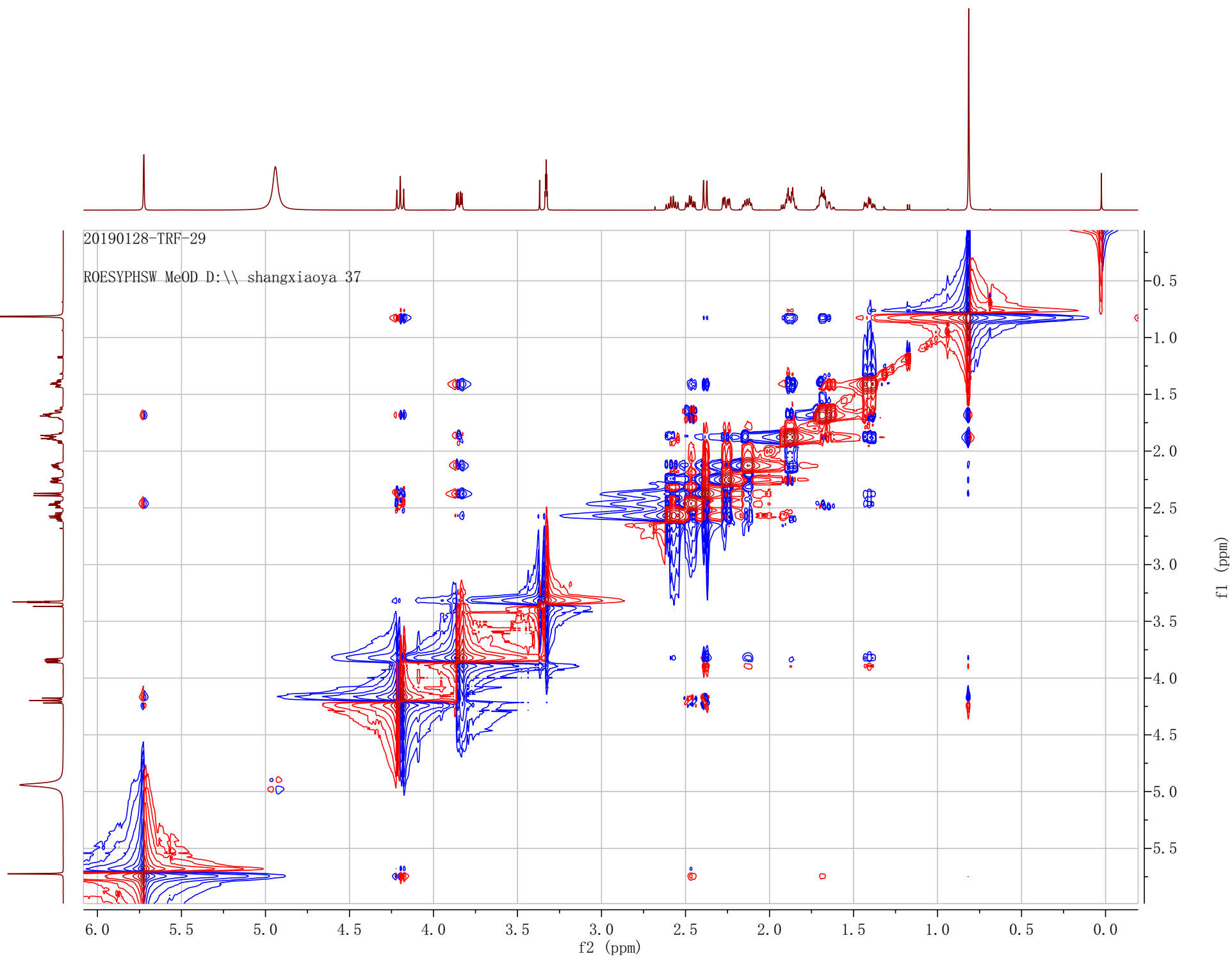

Supplement: Supplementary file 1 [file molecules-24-01701-s001.zip › molecules-489408-proofreading done-Suppl/data of compounds 1-10/Compound 1-NOE.pdf]

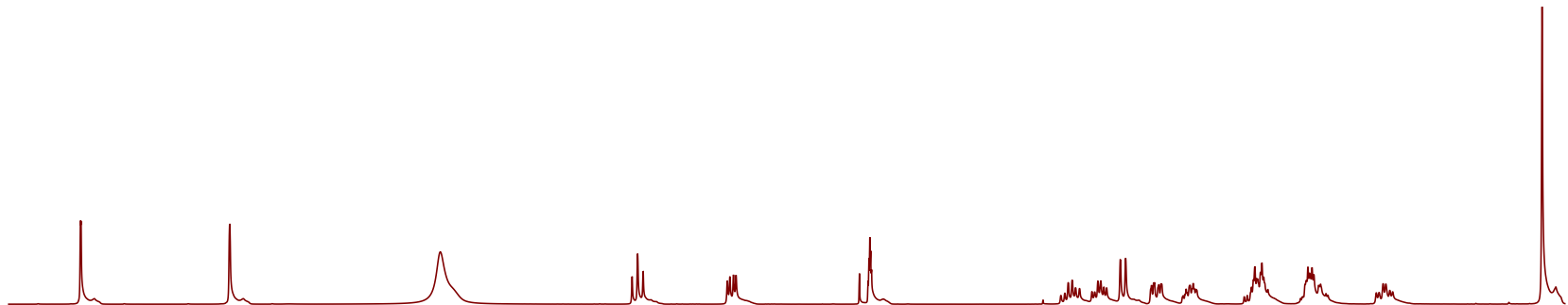

20171101-TRF-29  
BRUKER AV-III-500 COSY-NMR TRF-29 IN DMSO 2017.11.01  
HSQCETGPGSI DMSO D:\\ shangxiaoya 12

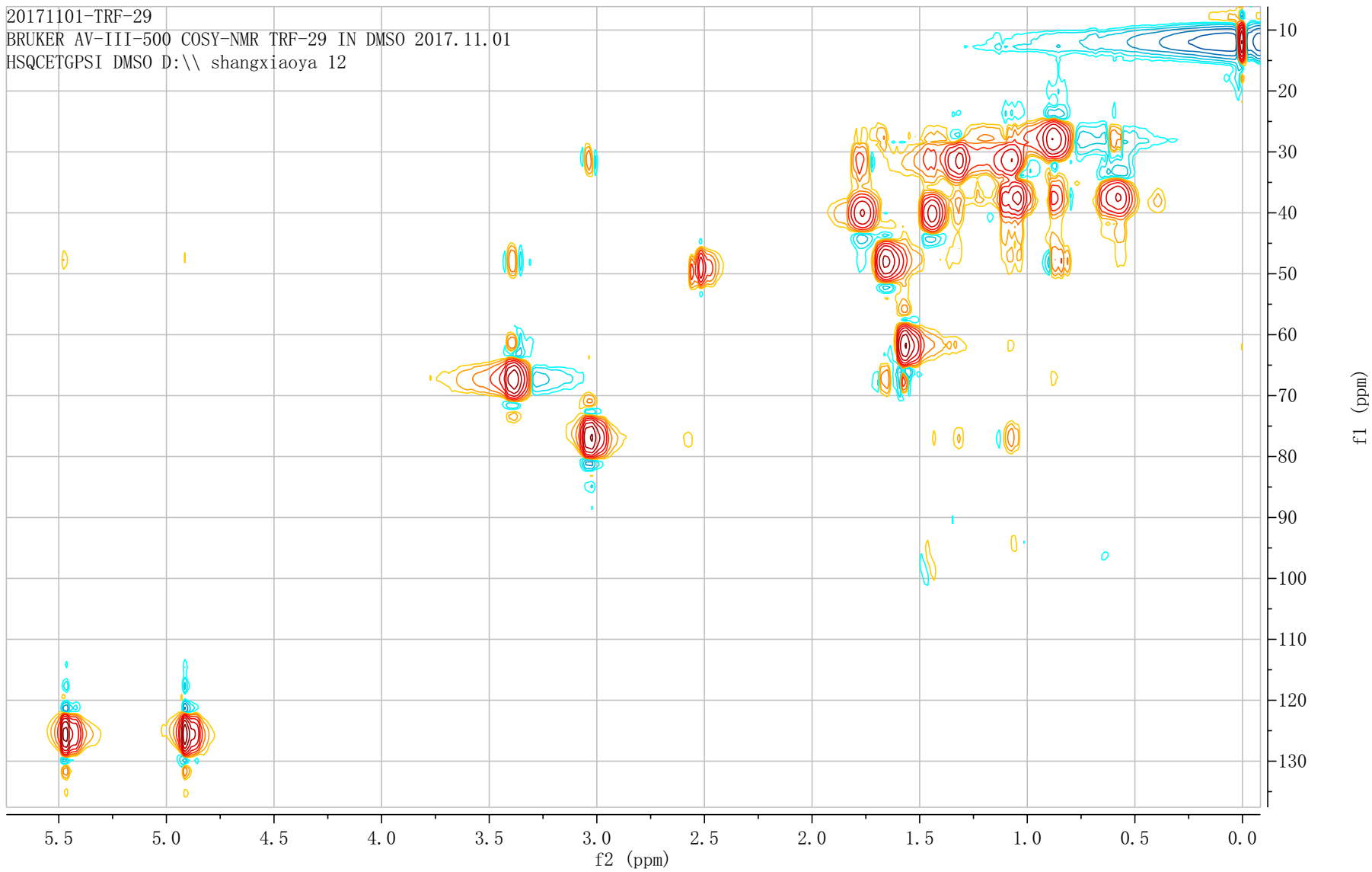

Supplement: Supplementary file 1 [file molecules-24-01701-s001.zip › molecules-489408-proofreading done-Suppl/data of compounds 1-10/Compound 1-QC.pdf]

20170908-TRF-28  
BRUKER AV-III-500 13C-NMR TRF-28  
13C-NMR DMSO D:\ shangxiaoya 2017.09.08

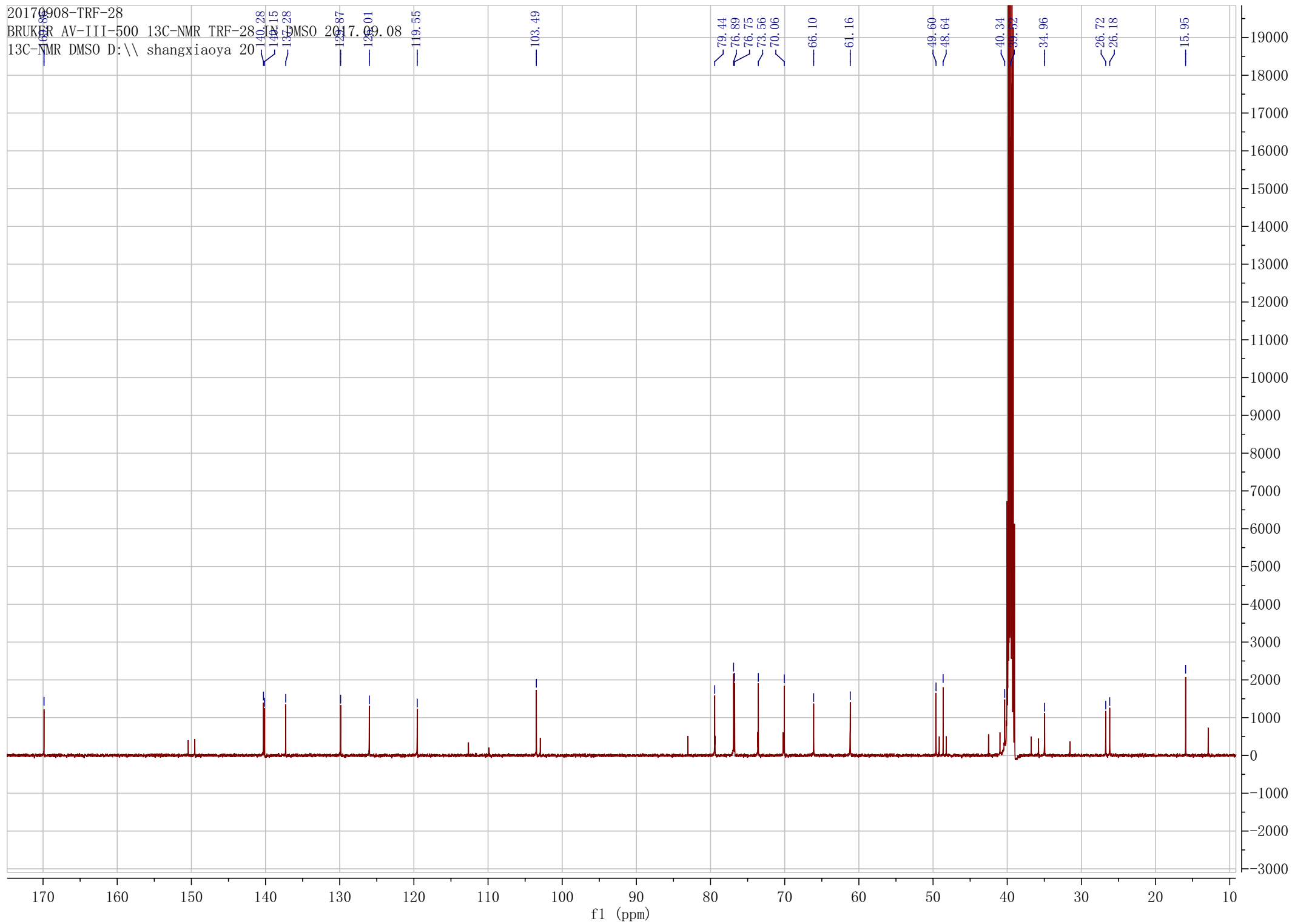

Supplement: Supplementary file 1 [file molecules-24-01701-s001.zip › molecules-489408-proofreading done-Suppl/data of compounds 1-10/Compound 10-C.pdf]

20170908-TRF-28  
BRUKER AV-III-500-1H-NMR TRF-28 IN DMSO-2017.09.08  
1H-NMR DMSO D:\shangxiaoya 20

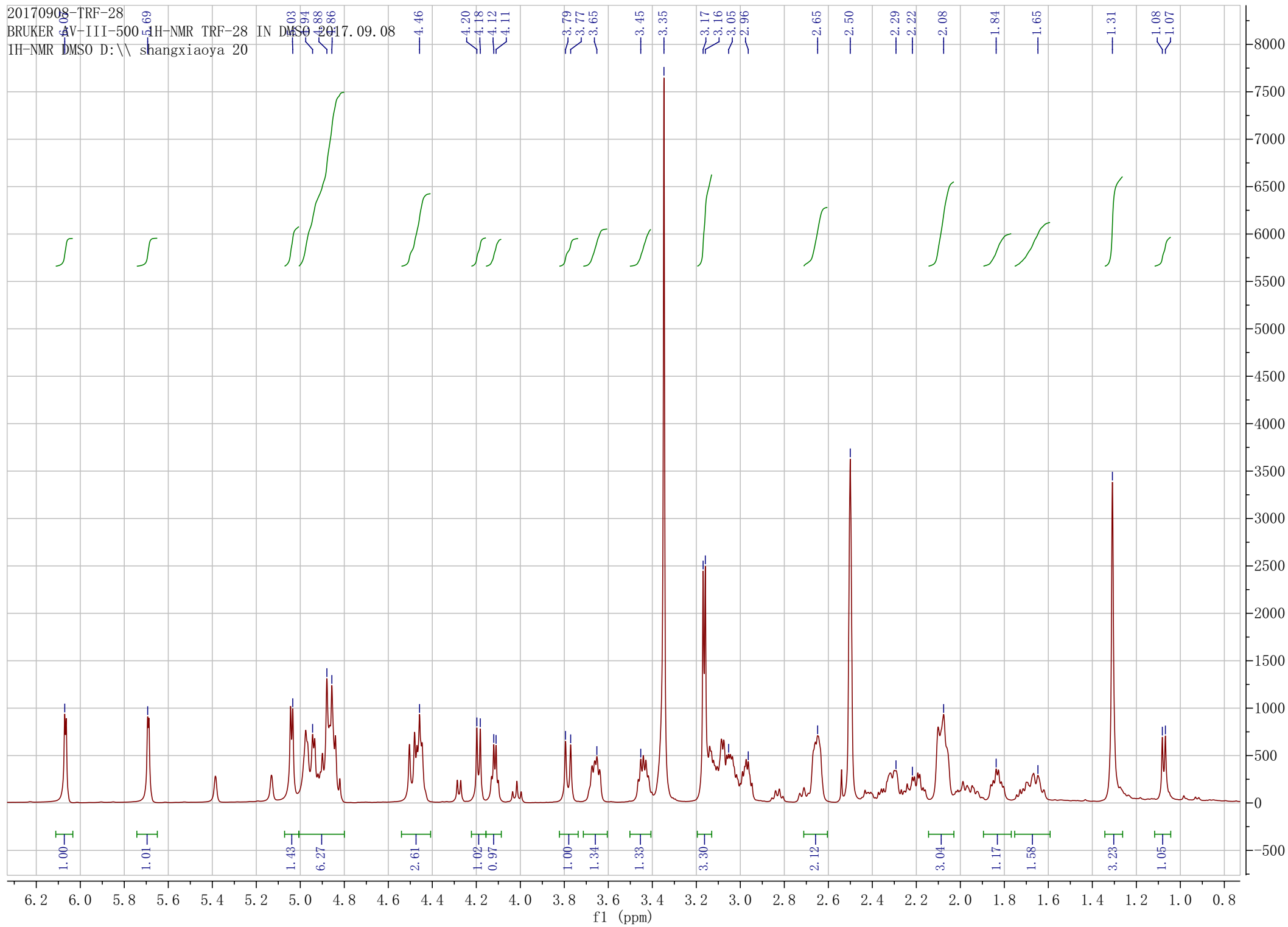

Supplement: Supplementary file 1 [file molecules-24-01701-s001.zip › molecules-489408-proofreading done-Suppl/data of compounds 1-10/Compound 10-H.pdf]

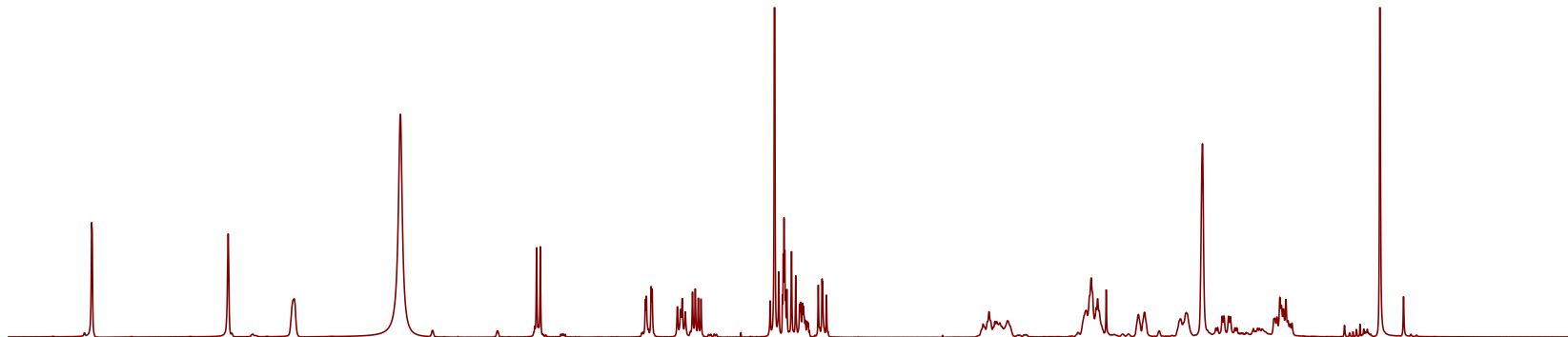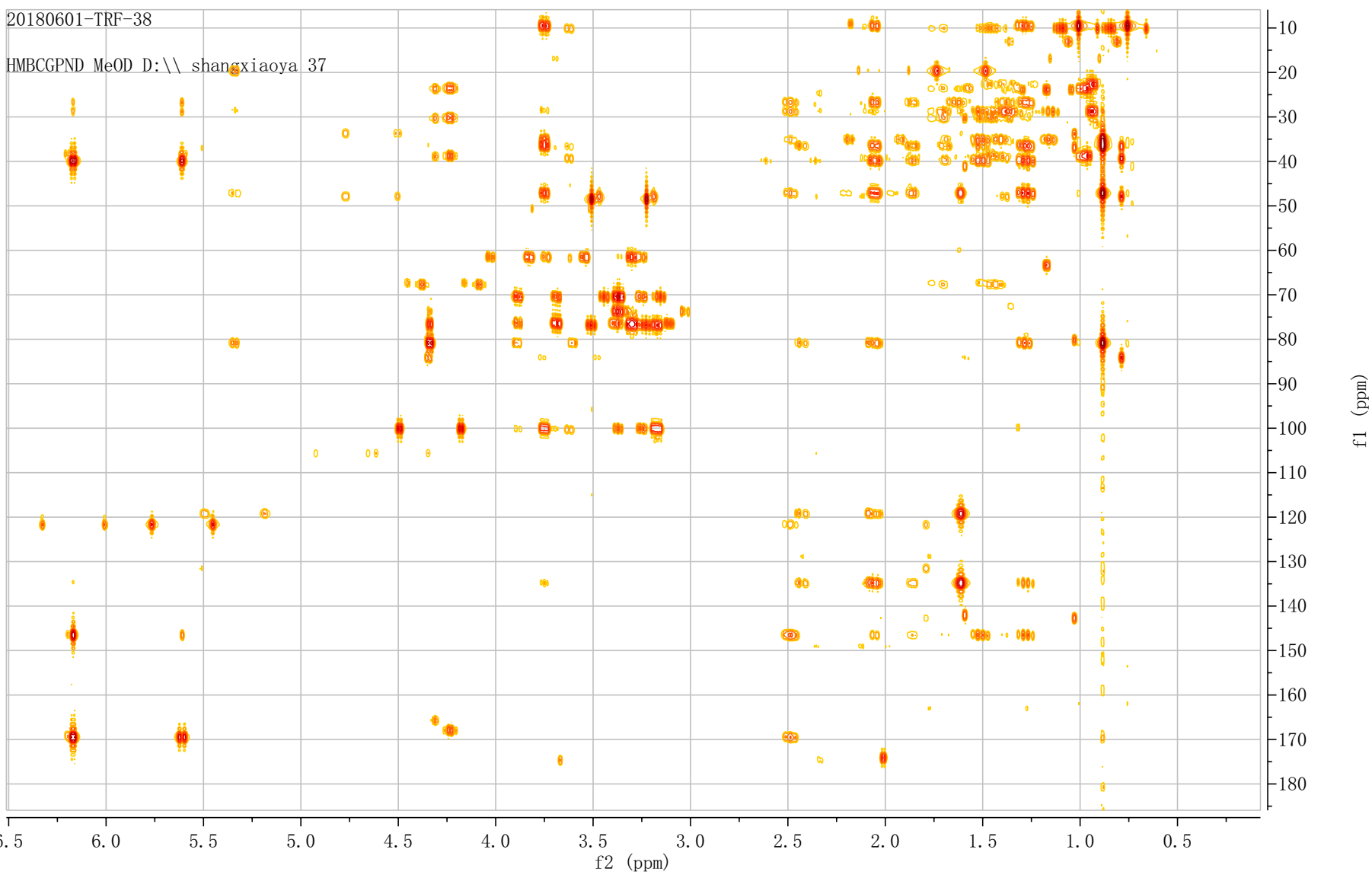

Supplement: Supplementary file 1 [file molecules-24-01701-s001.zip › molecules-489408-proofreading done-Suppl/data of compounds 1-10/Compound 2-BC.pdf]

20171218-TRF-38

<sup>13</sup>C-NMR MeOD D:\shangxiagya 7

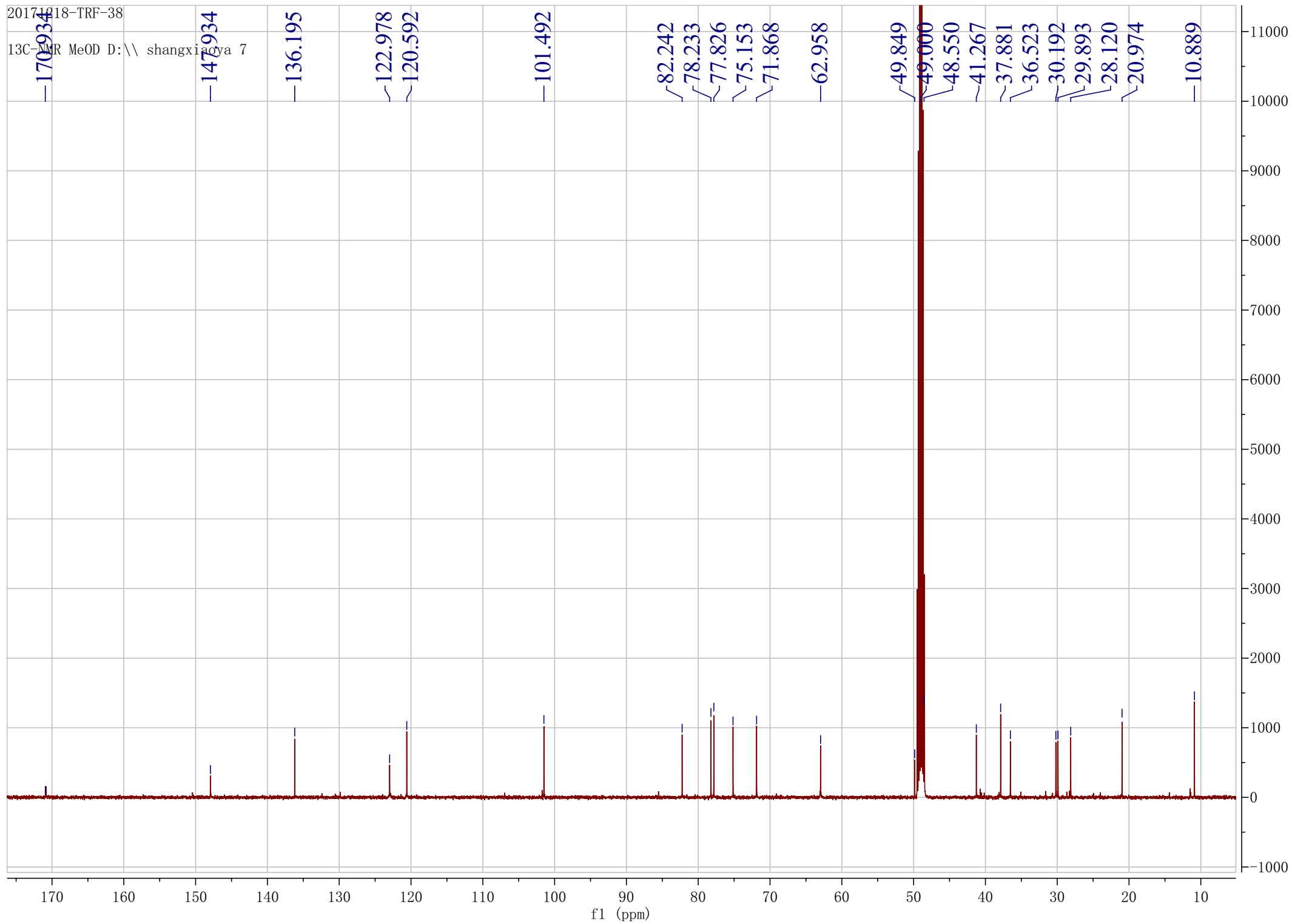

Supplement: Supplementary file 1 [file molecules-24-01701-s001.zip › molecules-489408-proofreading done-Suppl/data of compounds 1-10/Compound 2-C.pdf]

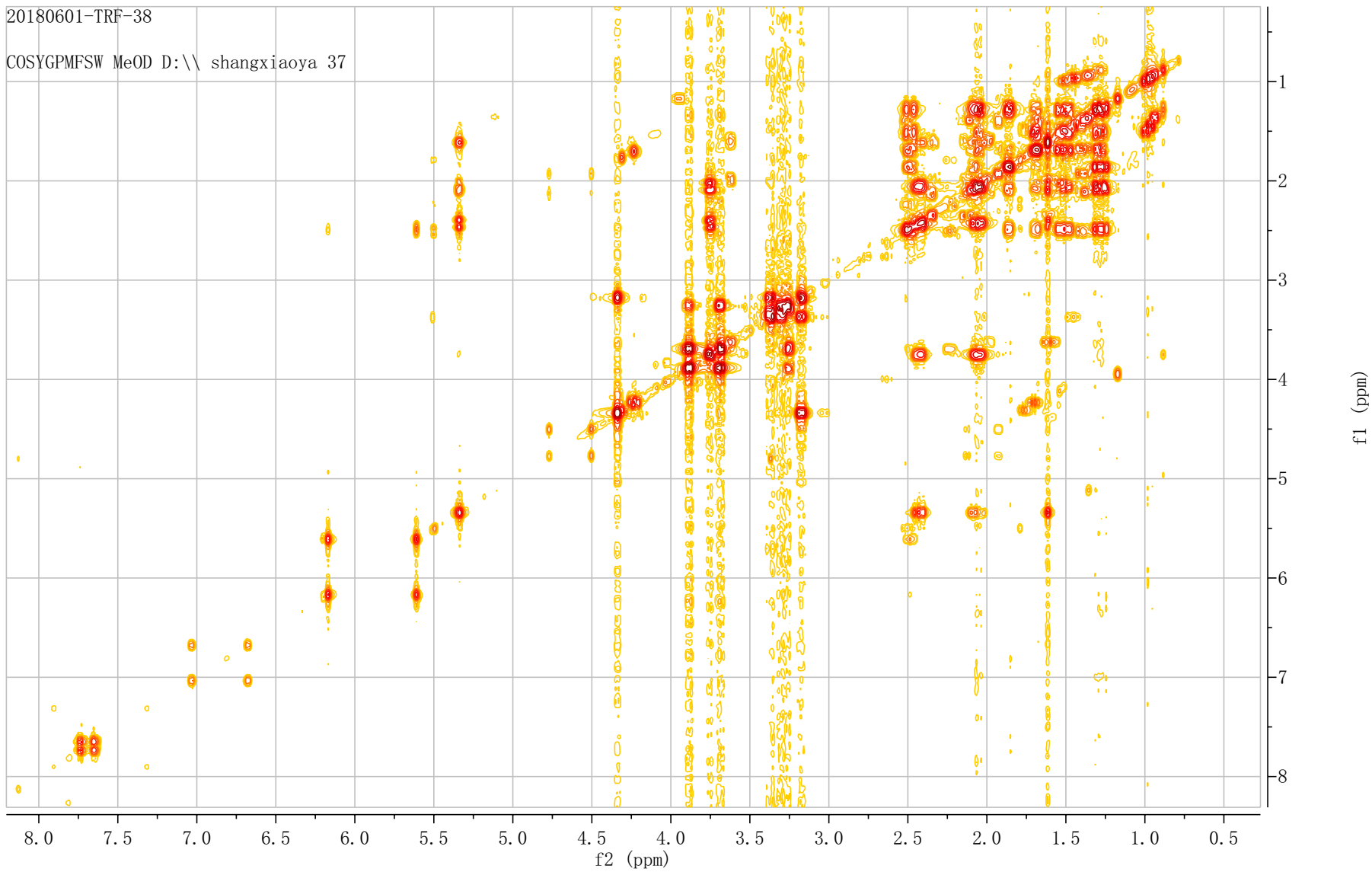

Supplement: Supplementary file 1 [file molecules-24-01701-s001.zip › molecules-489408-proofreading done-Suppl/data of compounds 1-10/Compound 2-COSY.pdf]

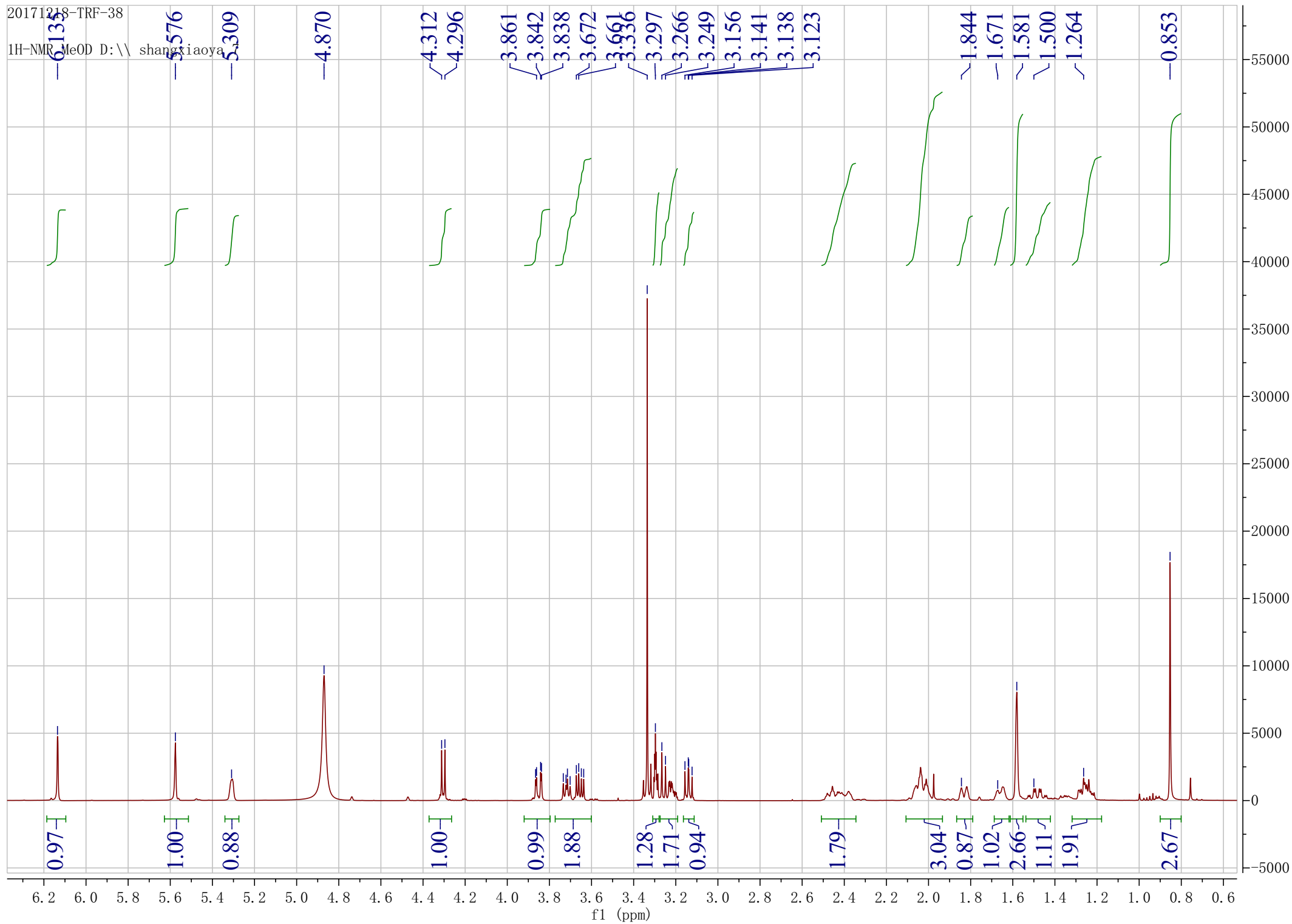

Supplement: Supplementary file 1 [file molecules-24-01701-s001.zip › molecules-489408-proofreading done-Suppl/data of compounds 1-10/Compound 2-H.pdf]

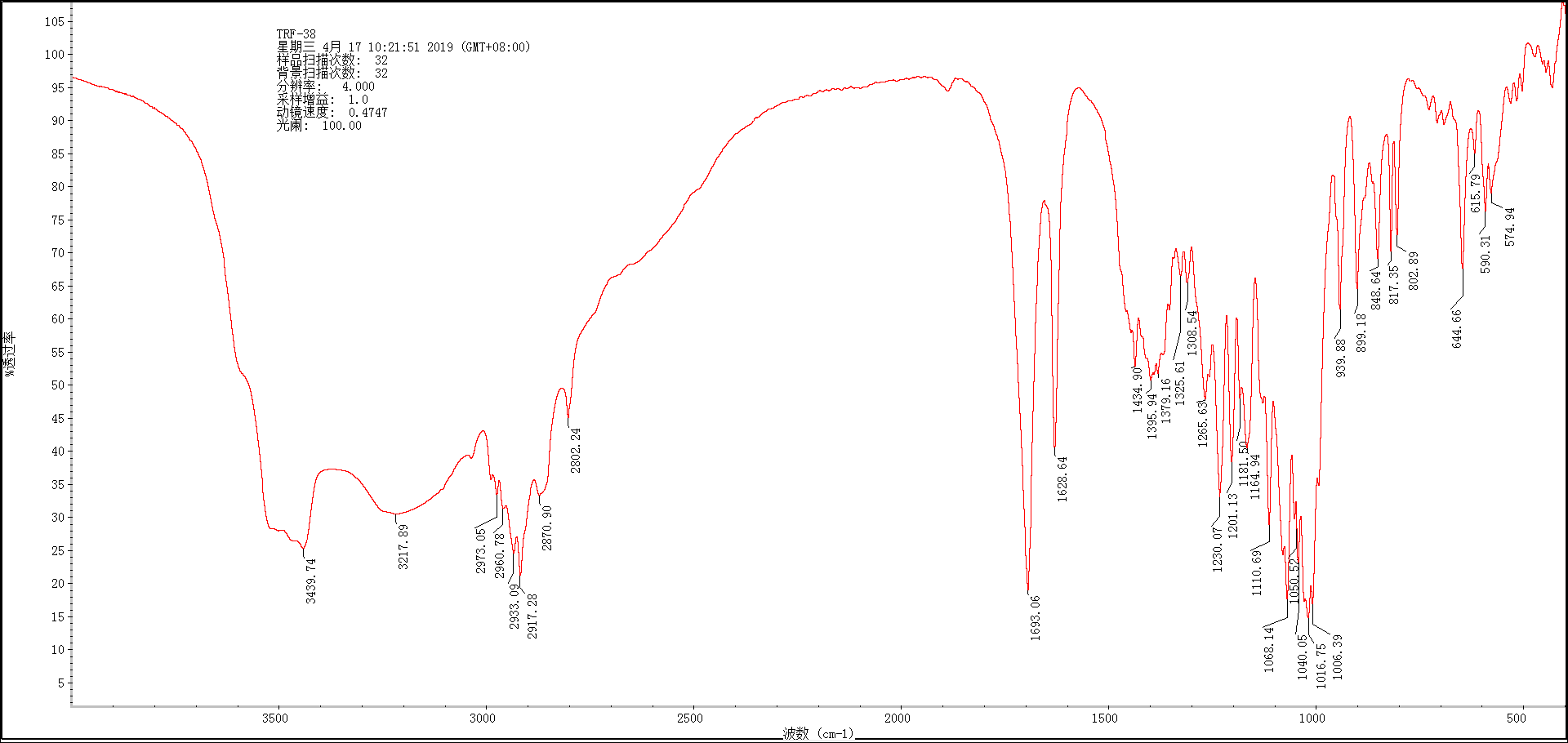

Supplement: Supplementary file 1 [file molecules-24-01701-s001.zip › molecules-489408-proofreading done-Suppl/data of compounds 1-10/Compound 2-IR.TIF]

TRF-38\_Pos\_FullMs #317 RT: 1.61 AV: 1 NL: 1.02E9  
T: FTMS + p ESI Full ms [50.0000-750.0000]

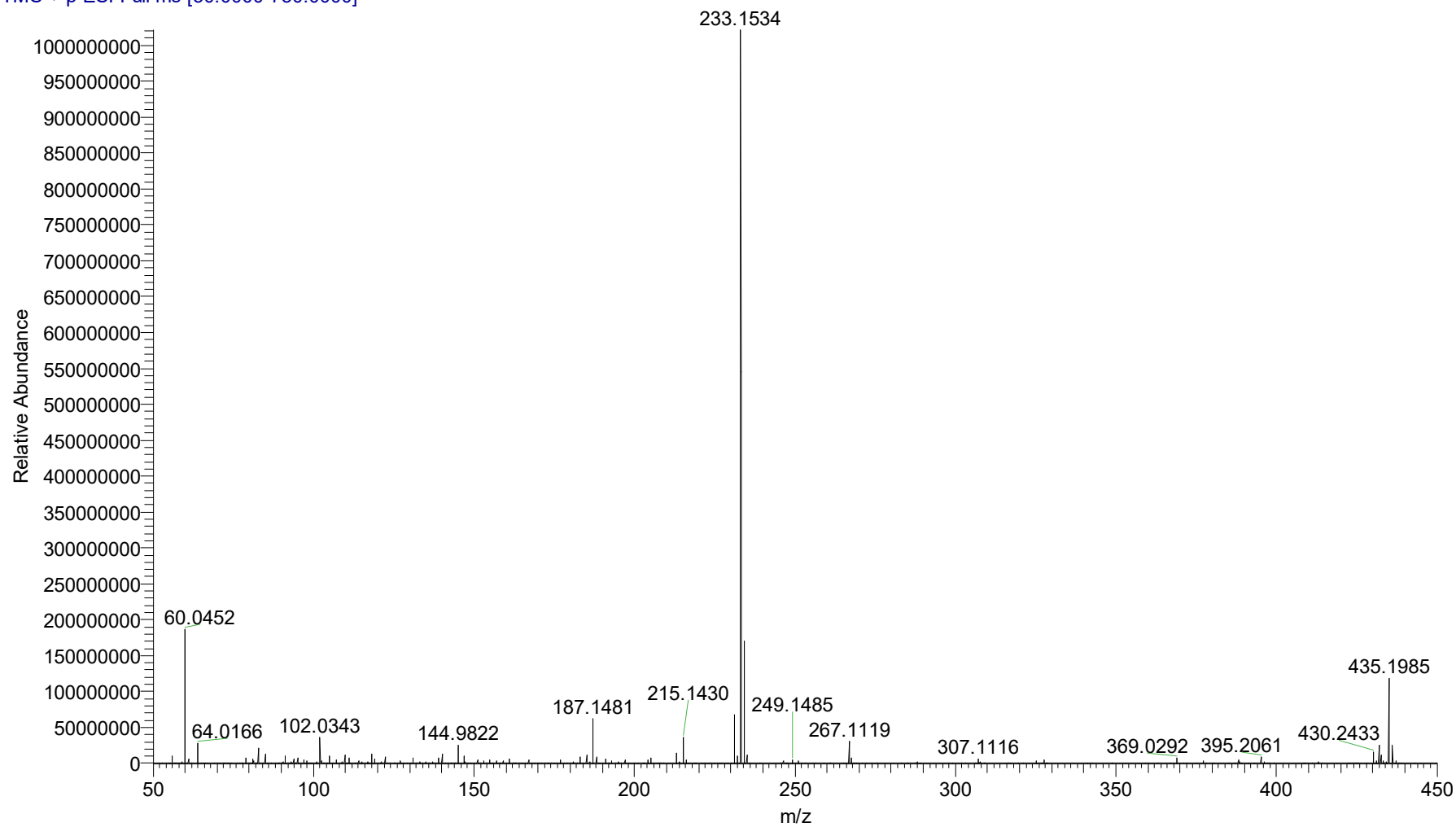

Supplement: Supplementary file 1 [file molecules-24-01701-s001.zip › molecules-489408-proofreading done-Suppl/data of compounds 1-10/Compound 2-MS.pdf]

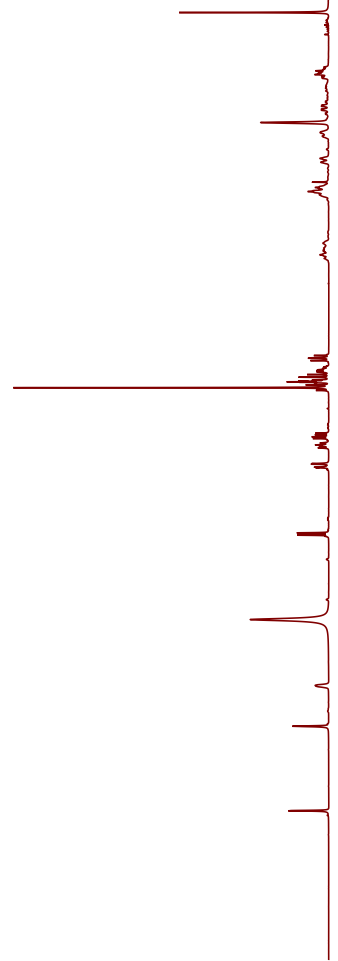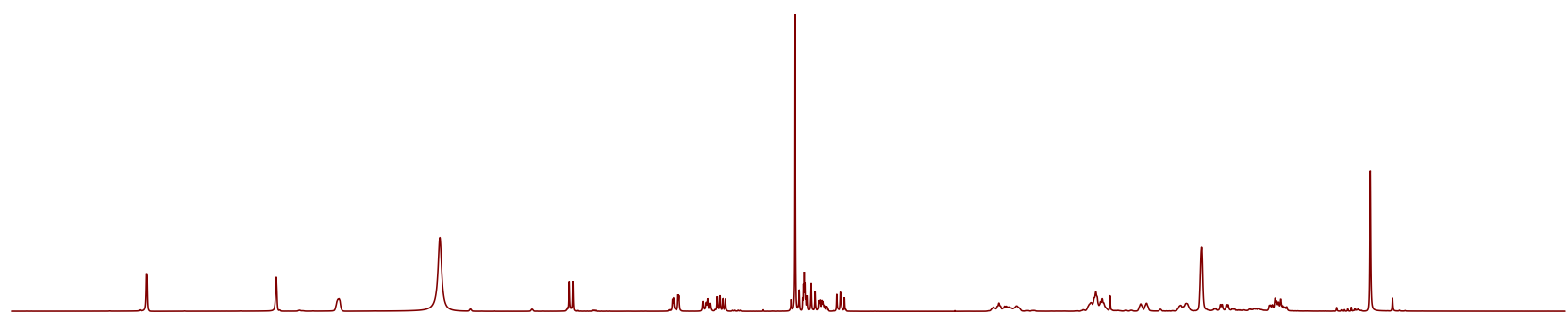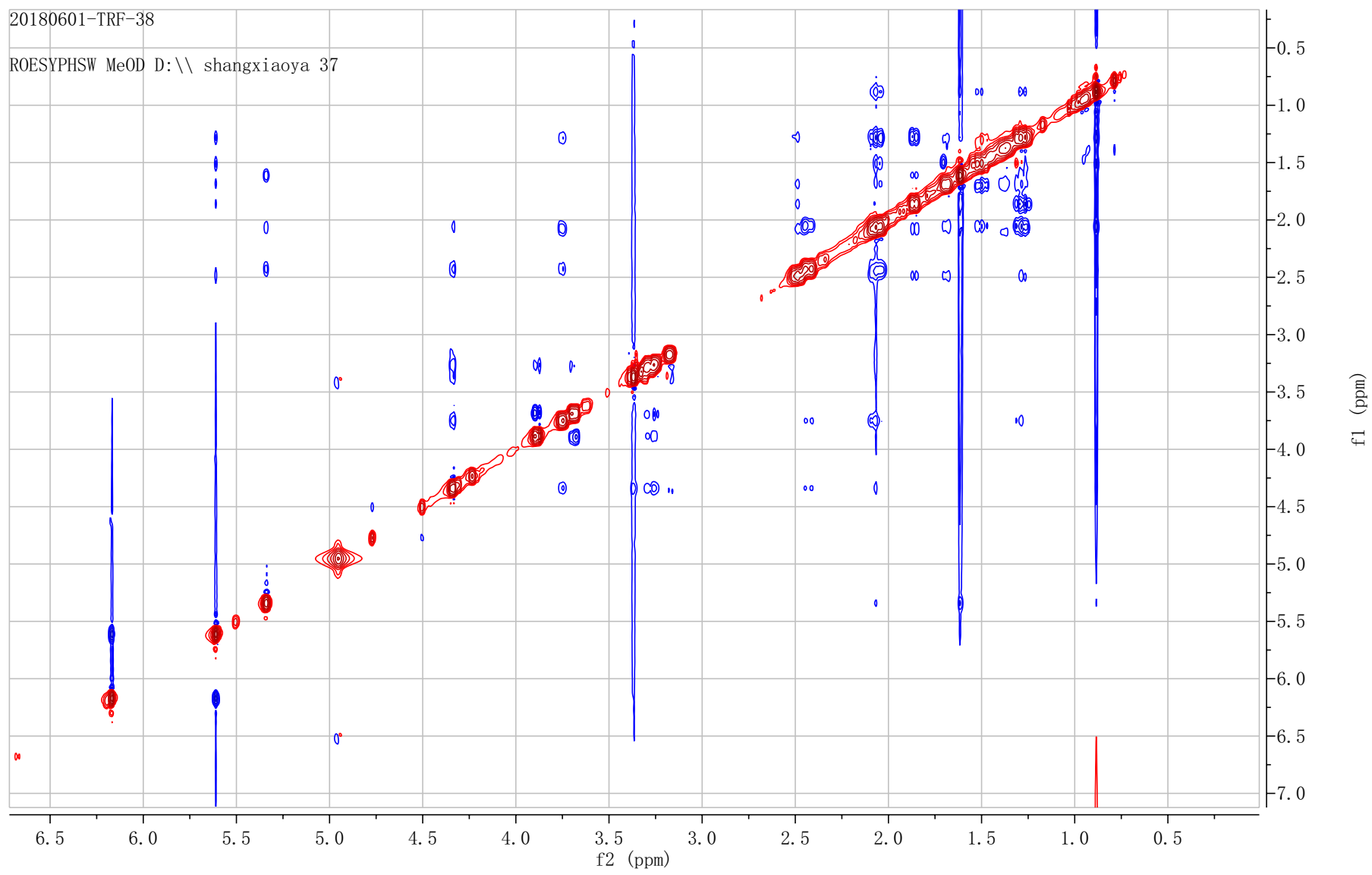

Supplement: Supplementary file 1 [file molecules-24-01701-s001.zip › molecules-489408-proofreading done-Suppl/data of compounds 1-10/Compound 2-NOE.pdf]

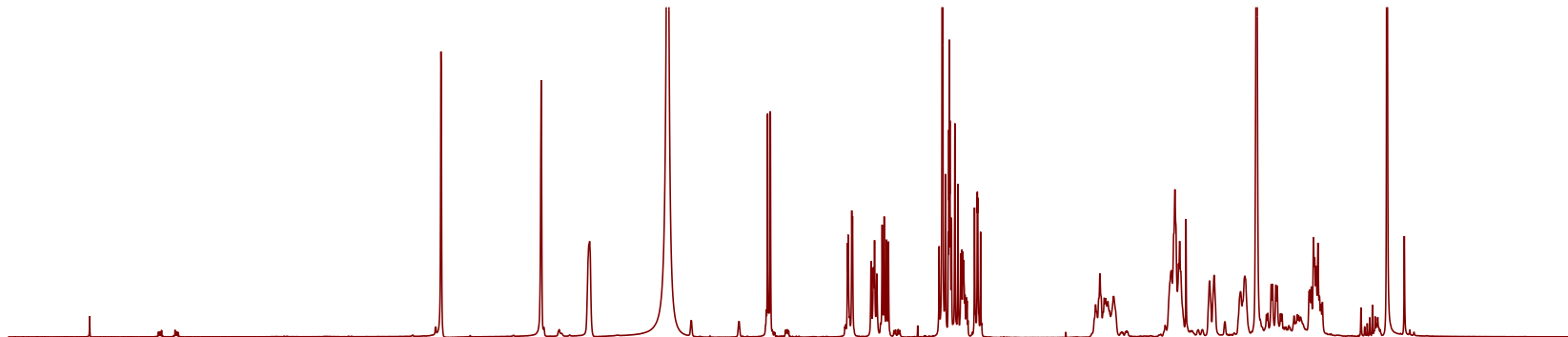

20180601-TRF-38

HSQCETGPSI MeOD D:\\ shangxiaoya 37

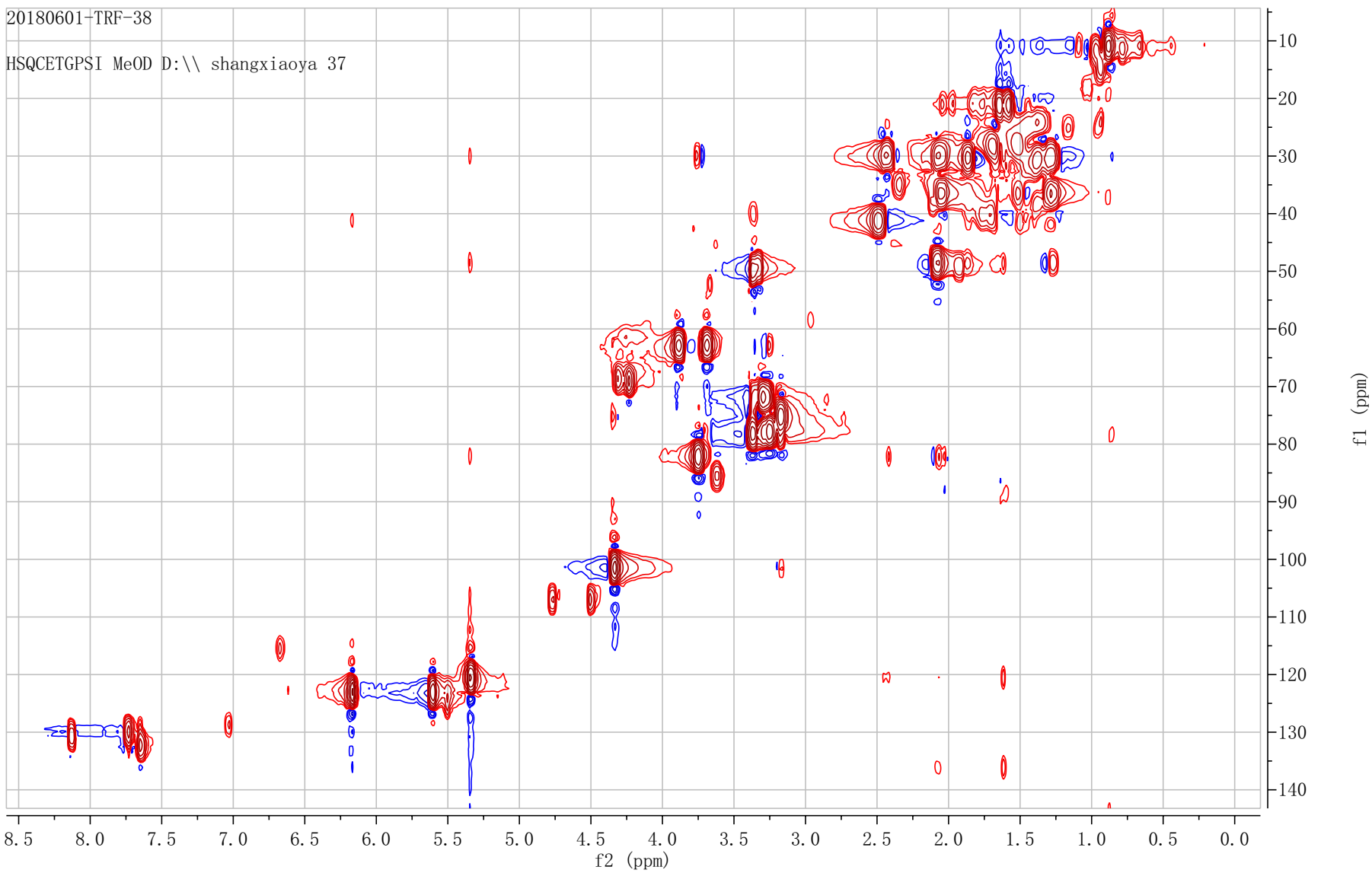

Supplement: Supplementary file 1 [file molecules-24-01701-s001.zip › molecules-489408-proofreading done-Suppl/data of compounds 1-10/Compound 2-QC.pdf]

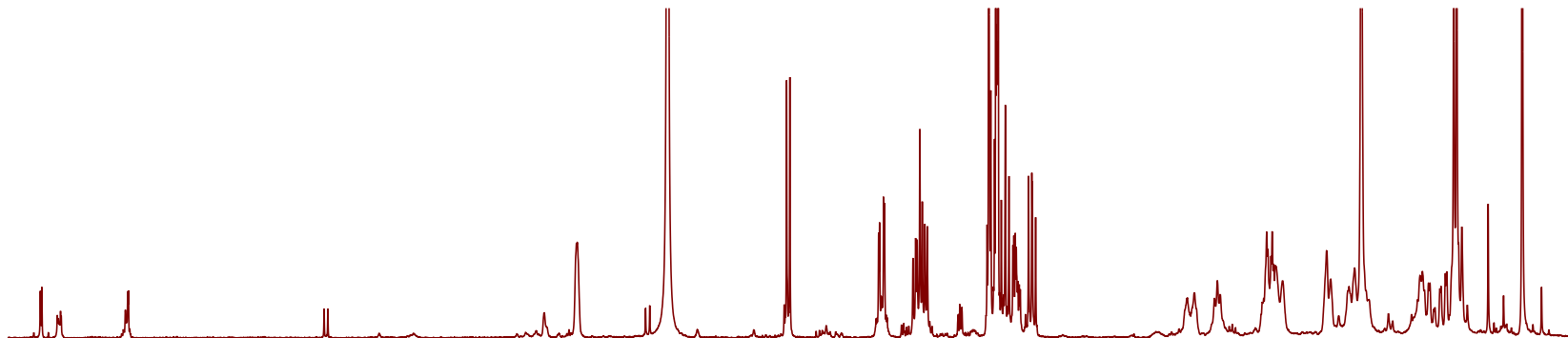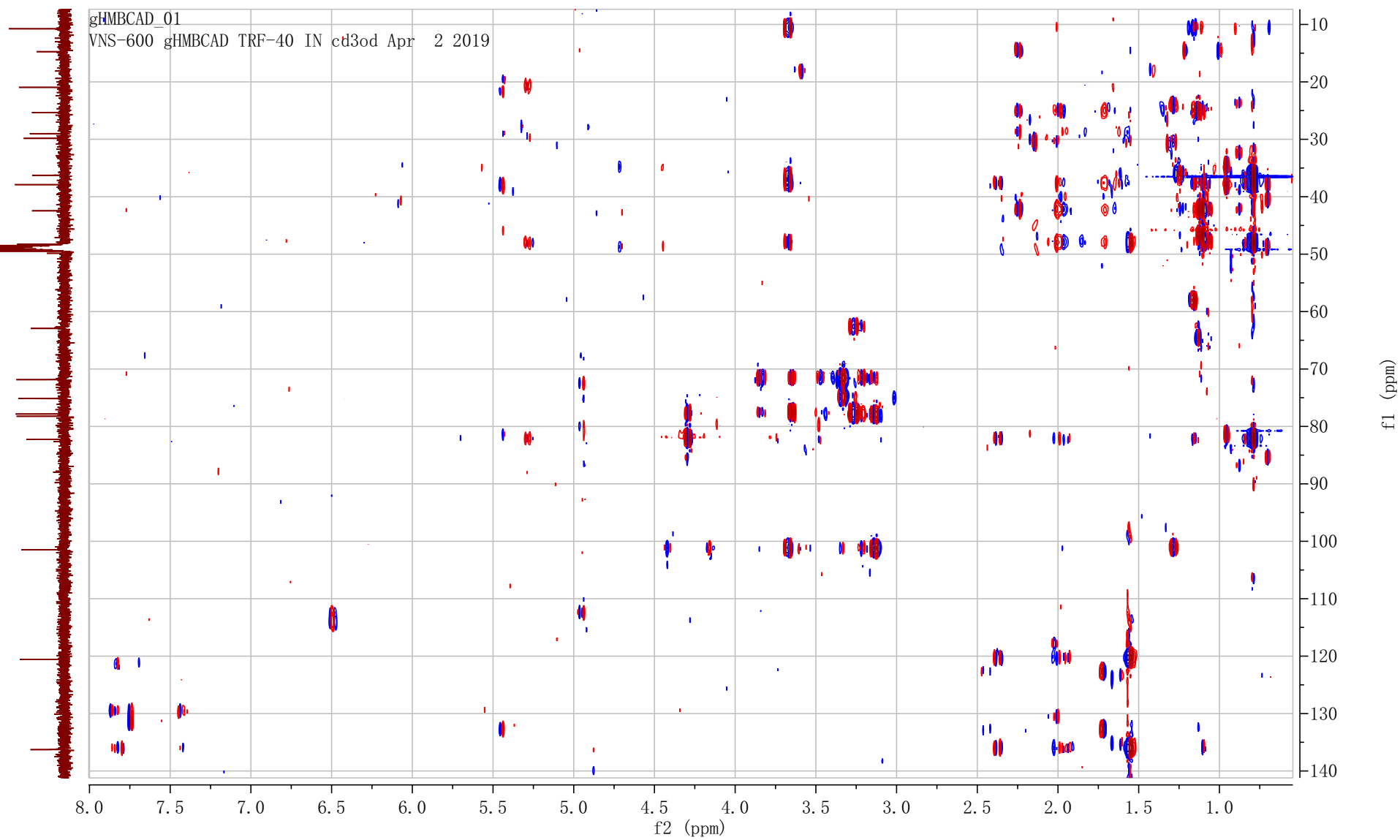

Supplement: Supplementary file 1 [file molecules-24-01701-s001.zip › molecules-489408-proofreading done-Suppl/data of compounds 1-10/Compound 3-BC.pdf]

20171218-TRF-40  
BRUKER AV-III-500 13C NMR TRF-40 IN CD3OD 2017.11.2  
13C NMR MeOD D:\\ shangxiaoya 17

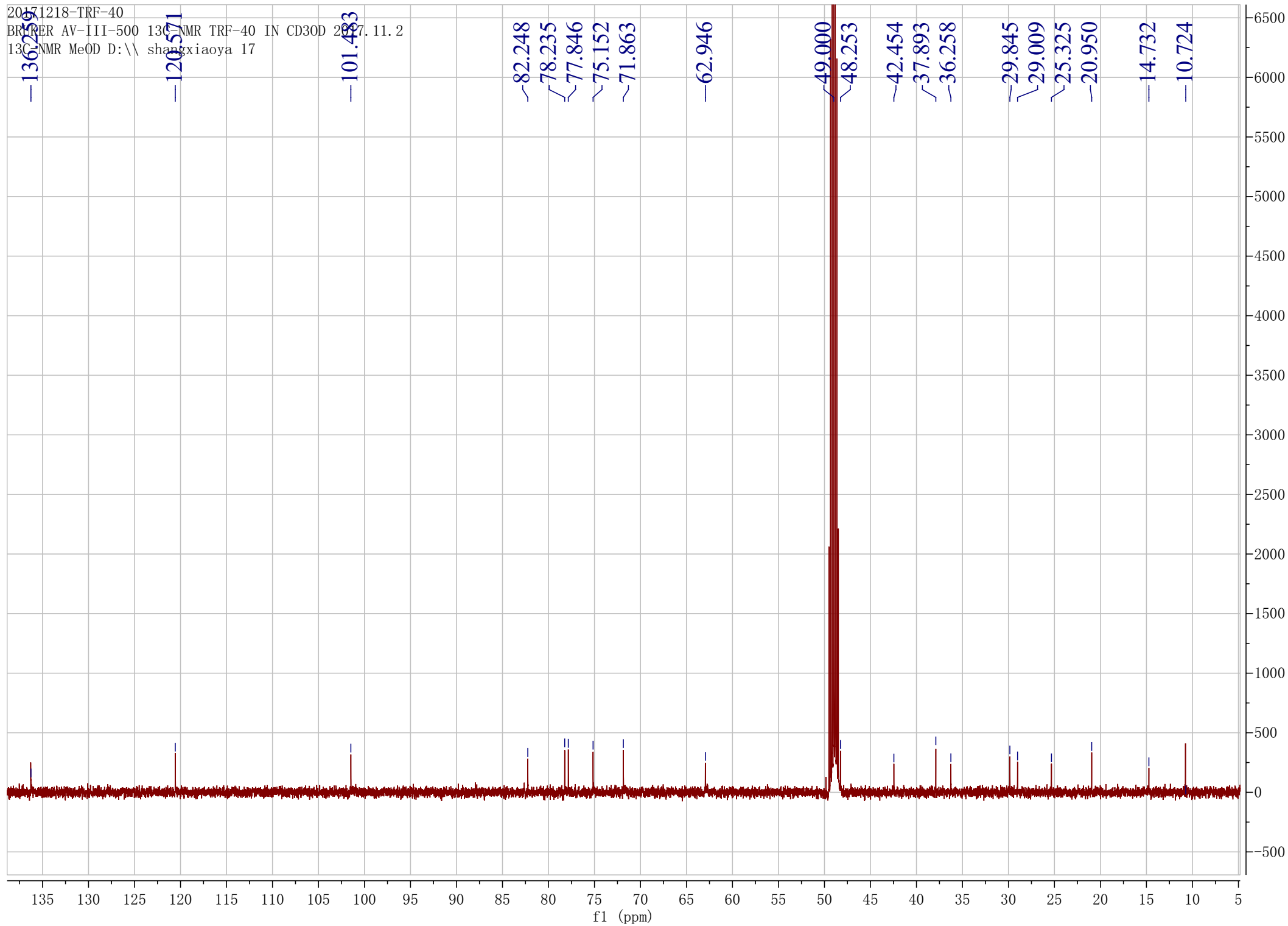

Supplement: Supplementary file 1 [file molecules-24-01701-s001.zip › molecules-489408-proofreading done-Suppl/data of compounds 1-10/Compound 3-C.pdf]

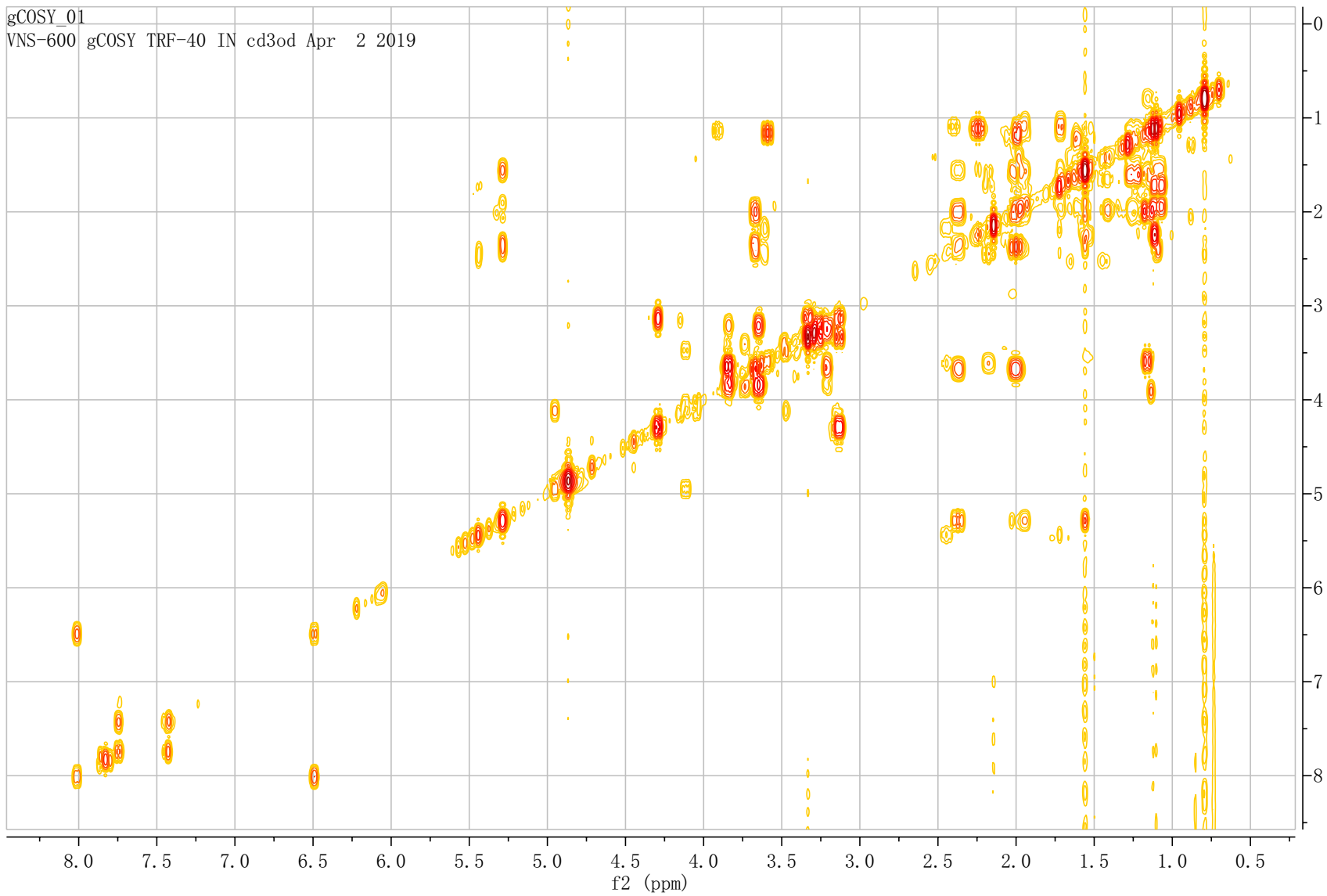

Supplement: Supplementary file 1 [file molecules-24-01701-s001.zip › molecules-489408-proofreading done-Suppl/data of compounds 1-10/Compound 3-COSY.pdf]

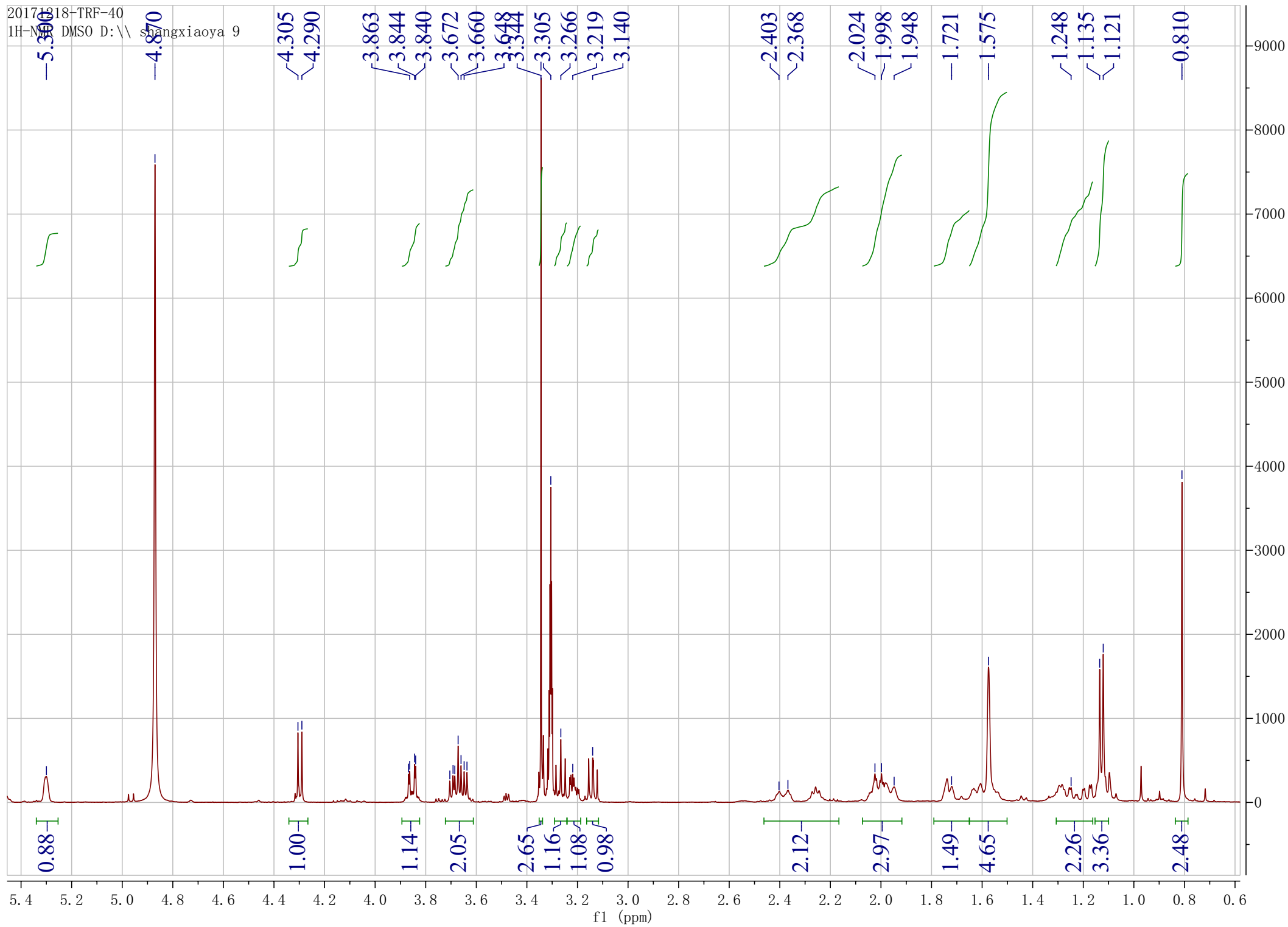

Supplement: Supplementary file 1 [file molecules-24-01701-s001.zip › molecules-489408-proofreading done-Suppl/data of compounds 1-10/Compound 3-H.pdf]

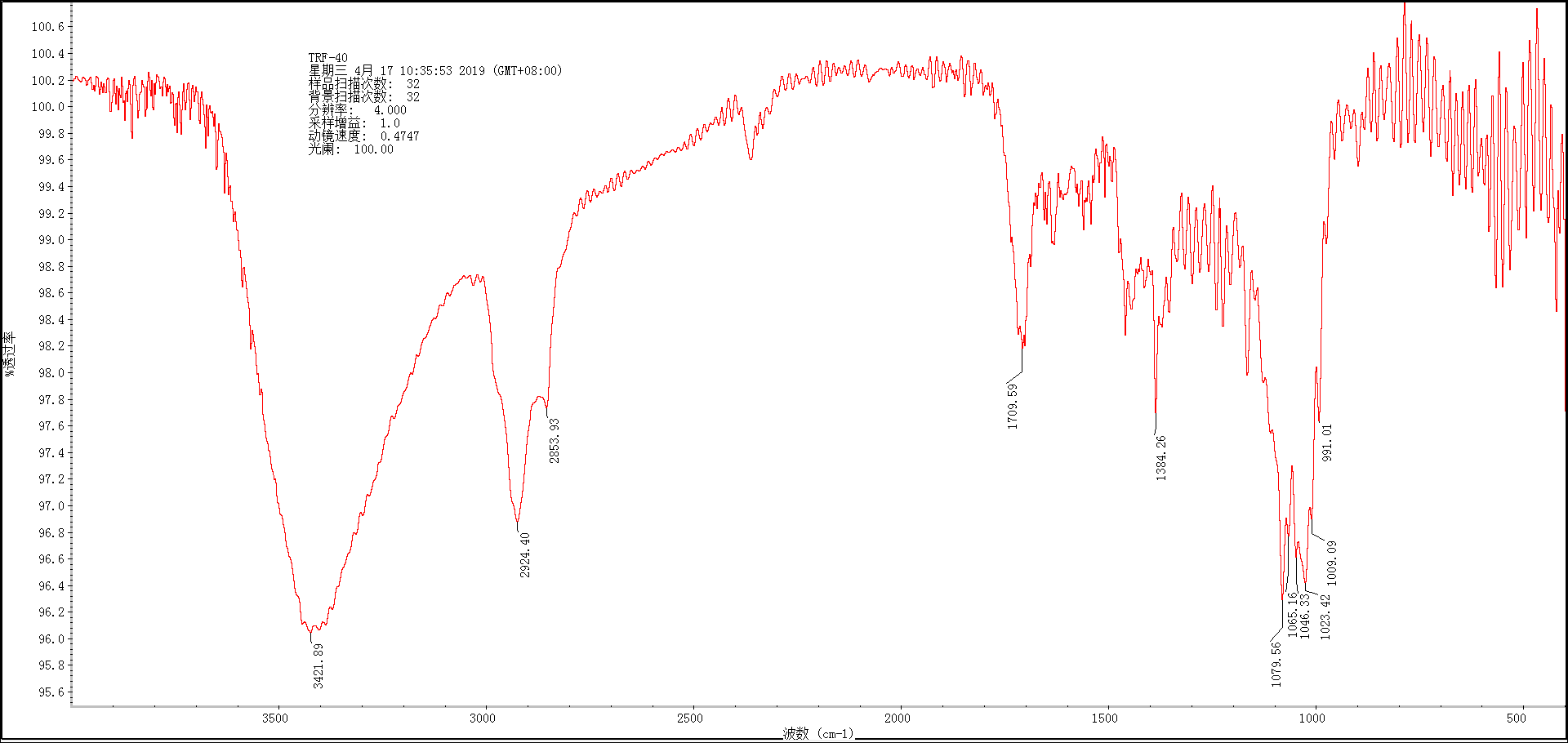

Supplement: Supplementary file 1 [file molecules-24-01701-s001.zip › molecules-489408-proofreading done-Suppl/data of compounds 1-10/Compound 3-IR.TIF]

TRF-40\_HC\_Pos\_FullMS#1001 RT: 4.38 AV: 1 NL: 6.19E6  
T: FTMS + p ESI Full ms [100.0000-750.0000]

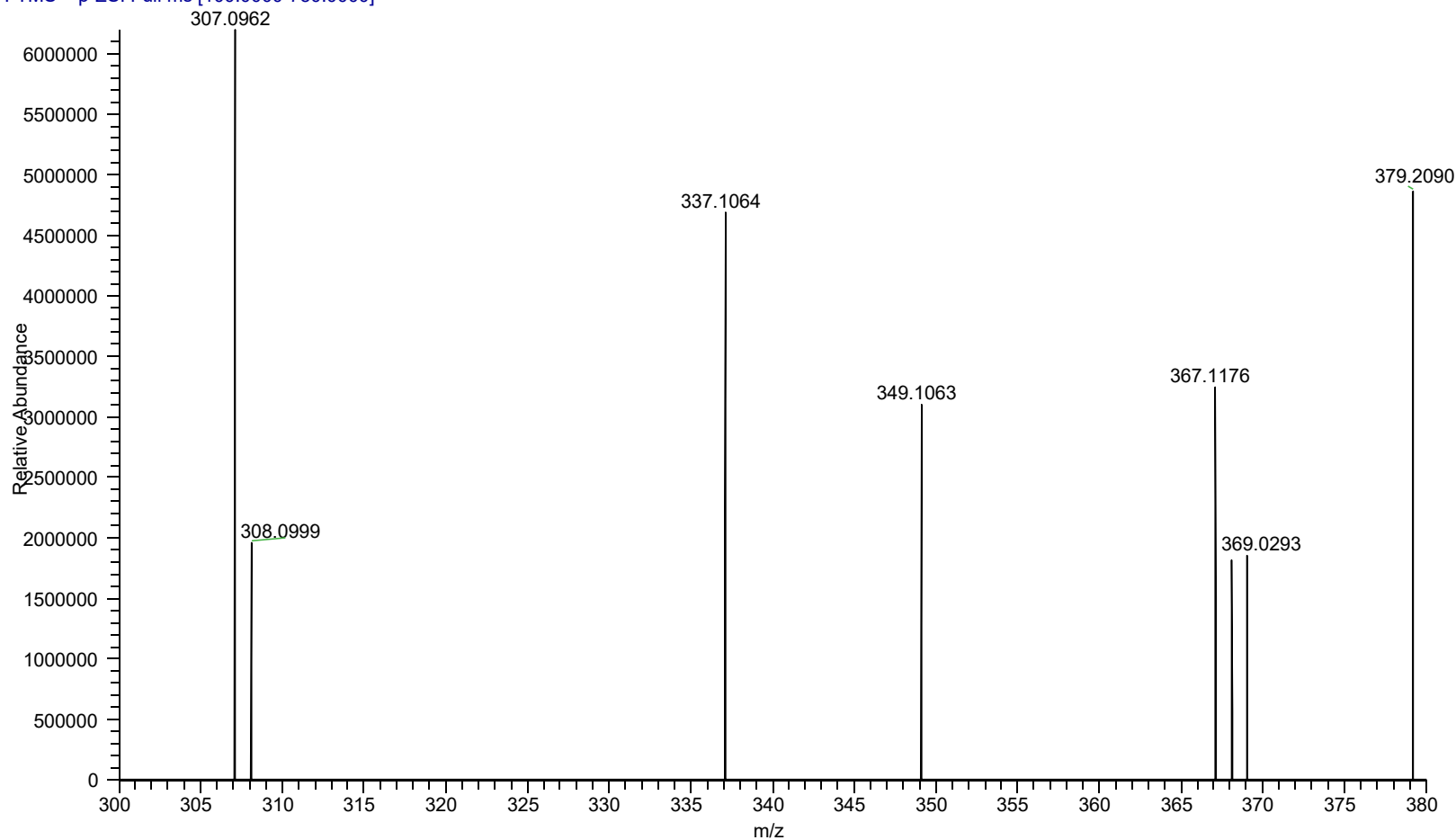

Supplement: Supplementary file 1 [file molecules-24-01701-s001.zip › molecules-489408-proofreading done-Suppl/data of compounds 1-10/Compound 3-MS.pdf]

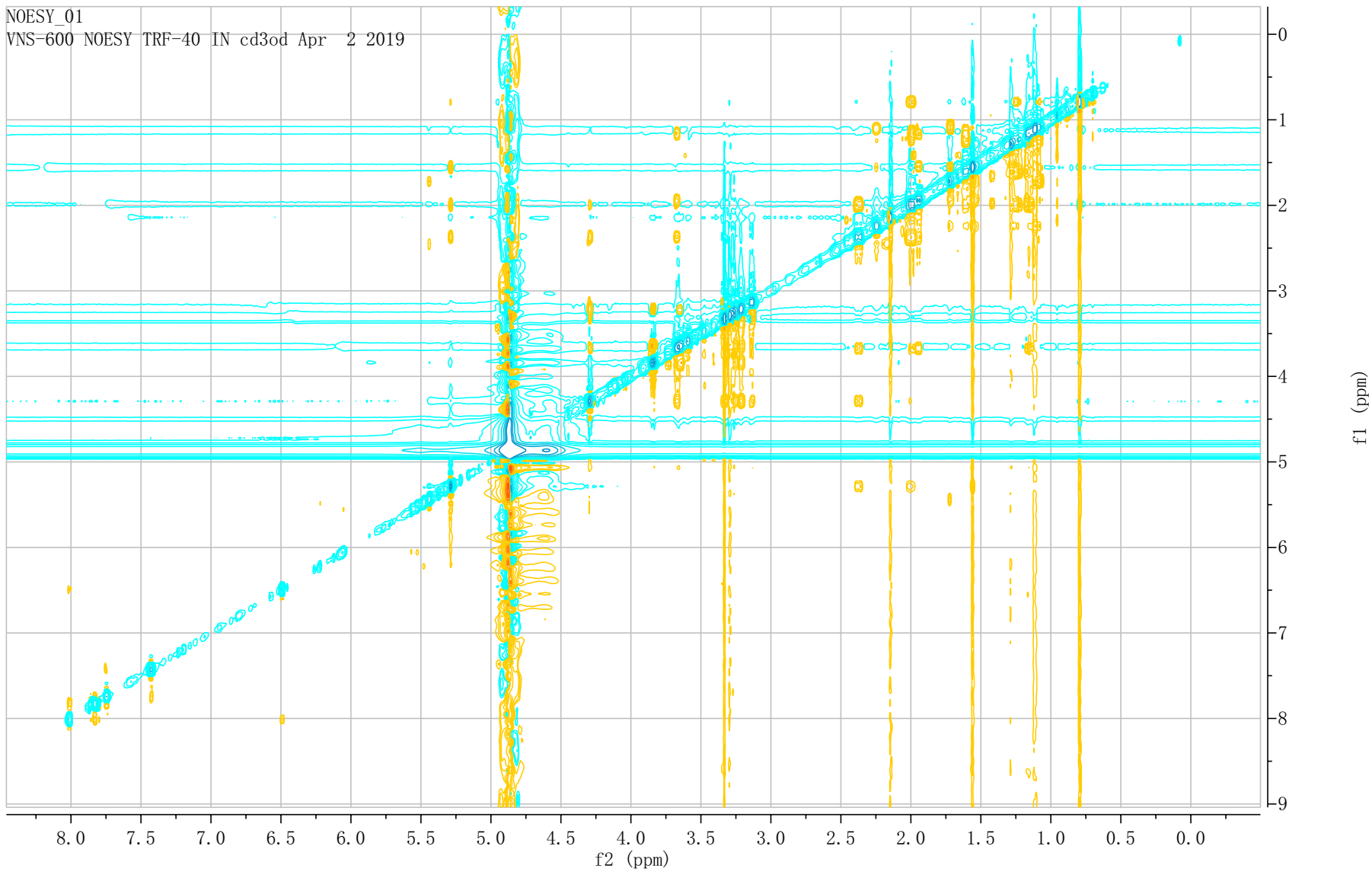

Supplement: Supplementary file 1 [file molecules-24-01701-s001.zip › molecules-489408-proofreading done-Suppl/data of compounds 1-10/Compound 3-NOE.pdf]

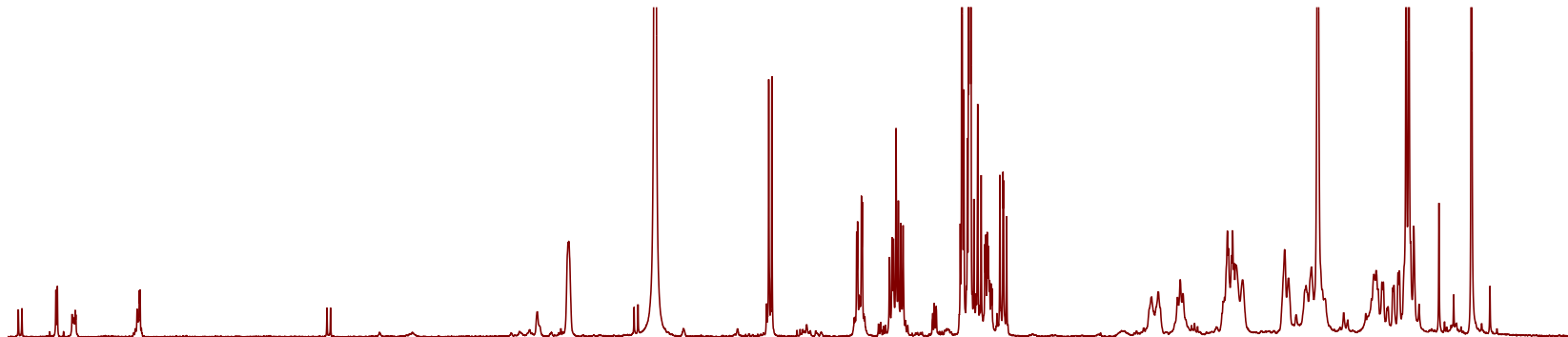

gHSQCAD\_01  
VNS-600 gHSQCAD TRF-40 IN cd3od Apr 2 2019

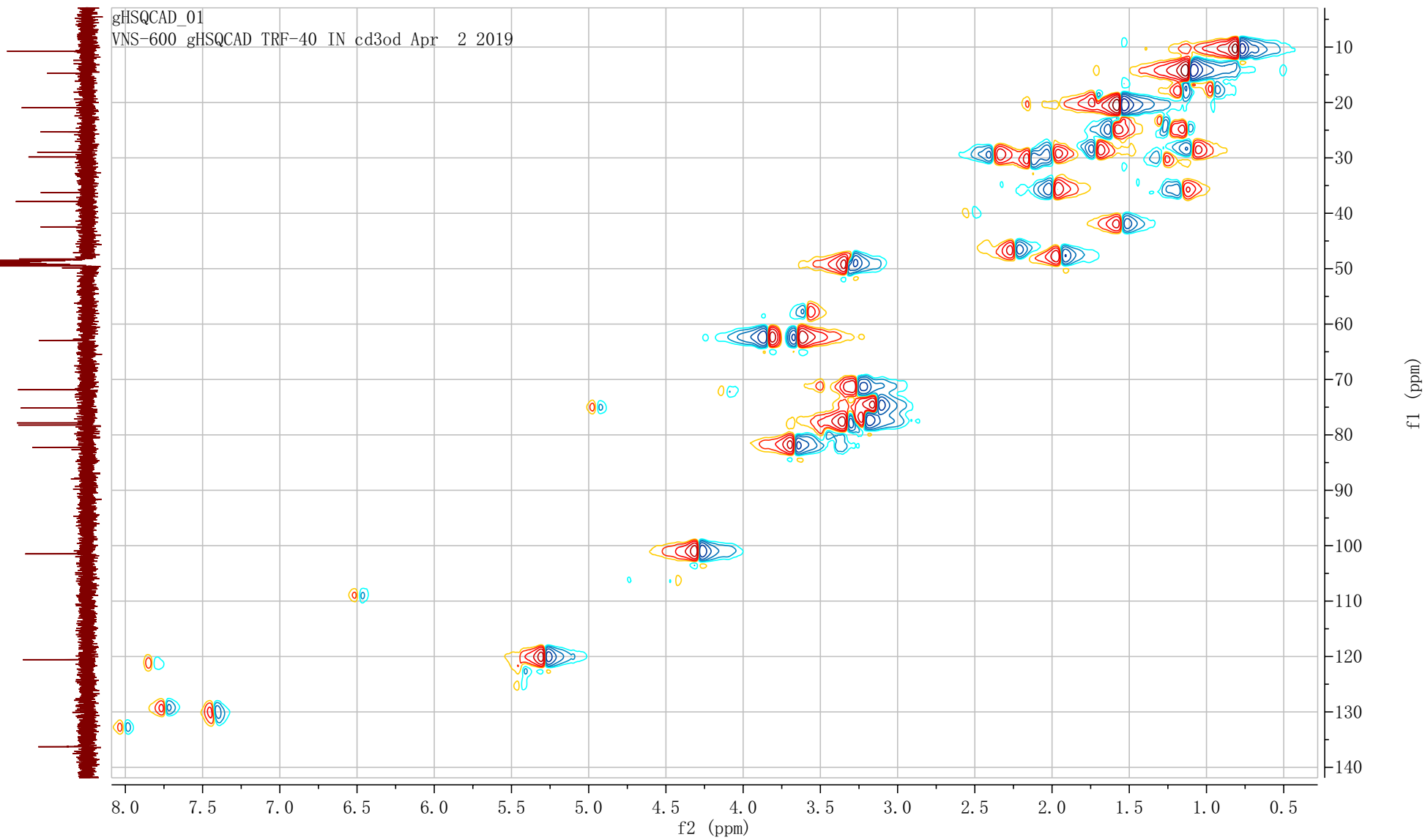

Supplement: Supplementary file 1 [file molecules-24-01701-s001.zip › molecules-489408-proofreading done-Suppl/data of compounds 1-10/Compound 3-QC.pdf]

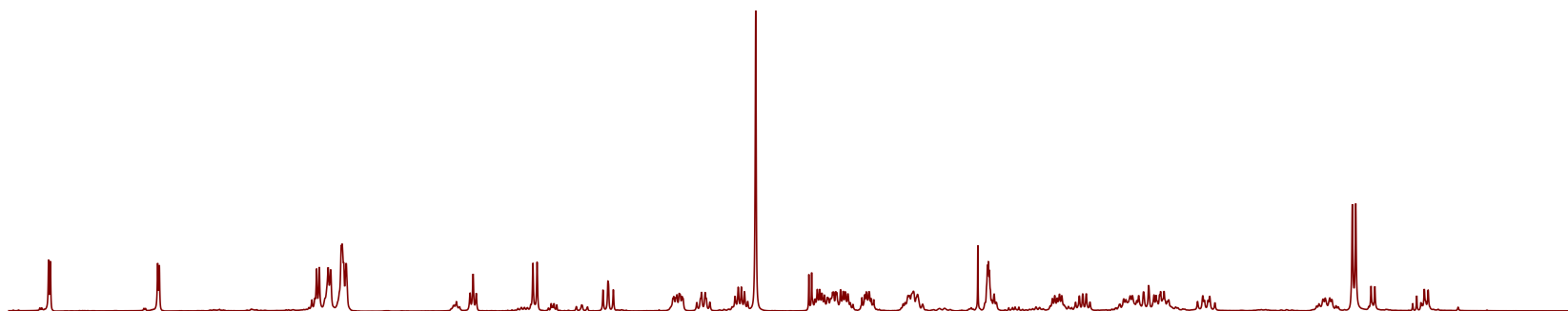

20171101-TRF-26  
BRUKER AV-III-500 COSY-NMR TRF-26 IN DMSO 2017.11.01  
HMBGPN DMSO D:\\ shangxiaoya 4

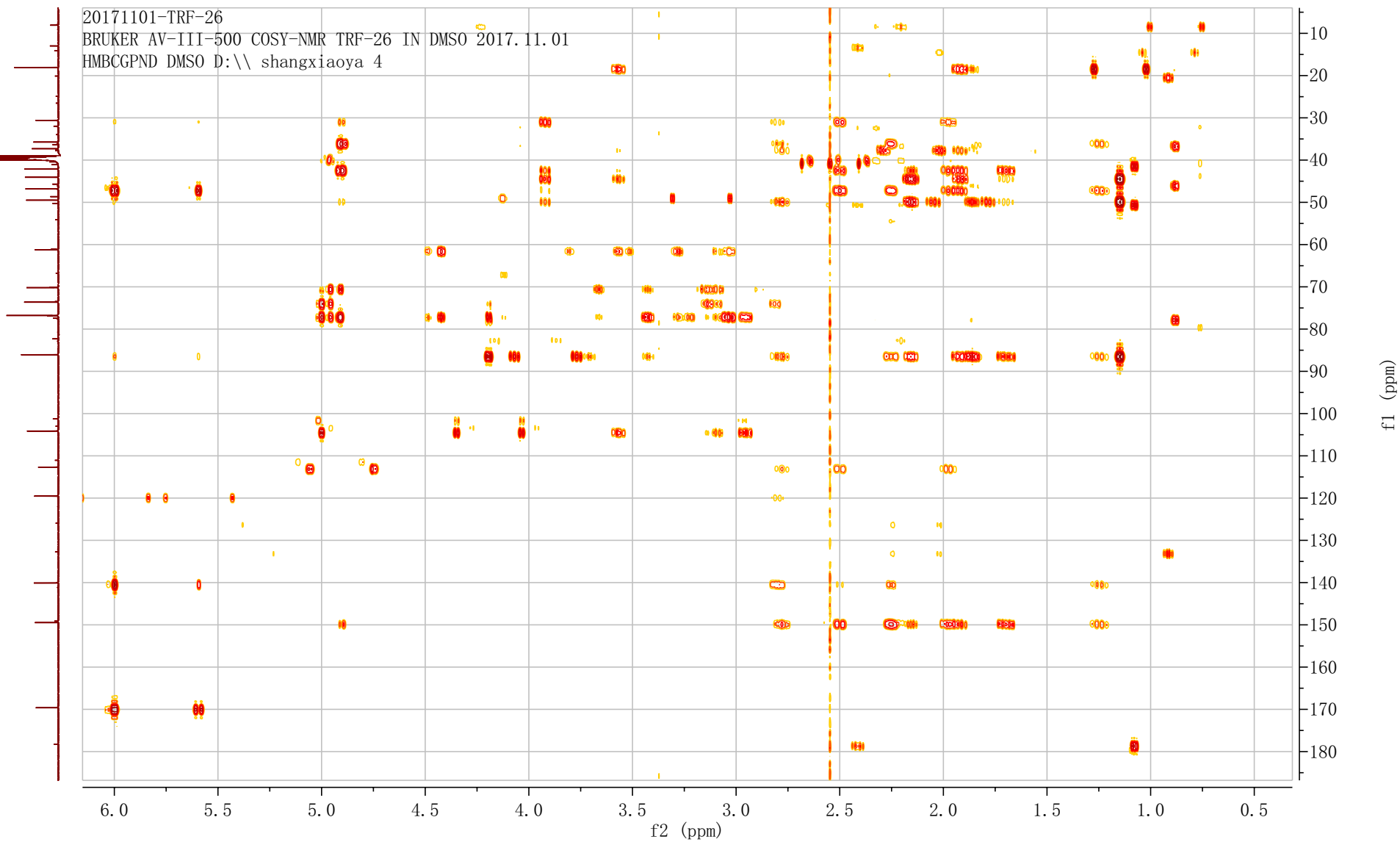

Supplement: Supplementary file 1 [file molecules-24-01701-s001.zip › molecules-489408-proofreading done-Suppl/data of compounds 1-10/Compound 4-BC.pdf]

20170908-TRF-26  
BRUKER AV-III-500 13C-NMR TRF-26 1N DMSO 2017.09.08  
13C-NMR DMSO D:\\ shangxiaoya 18

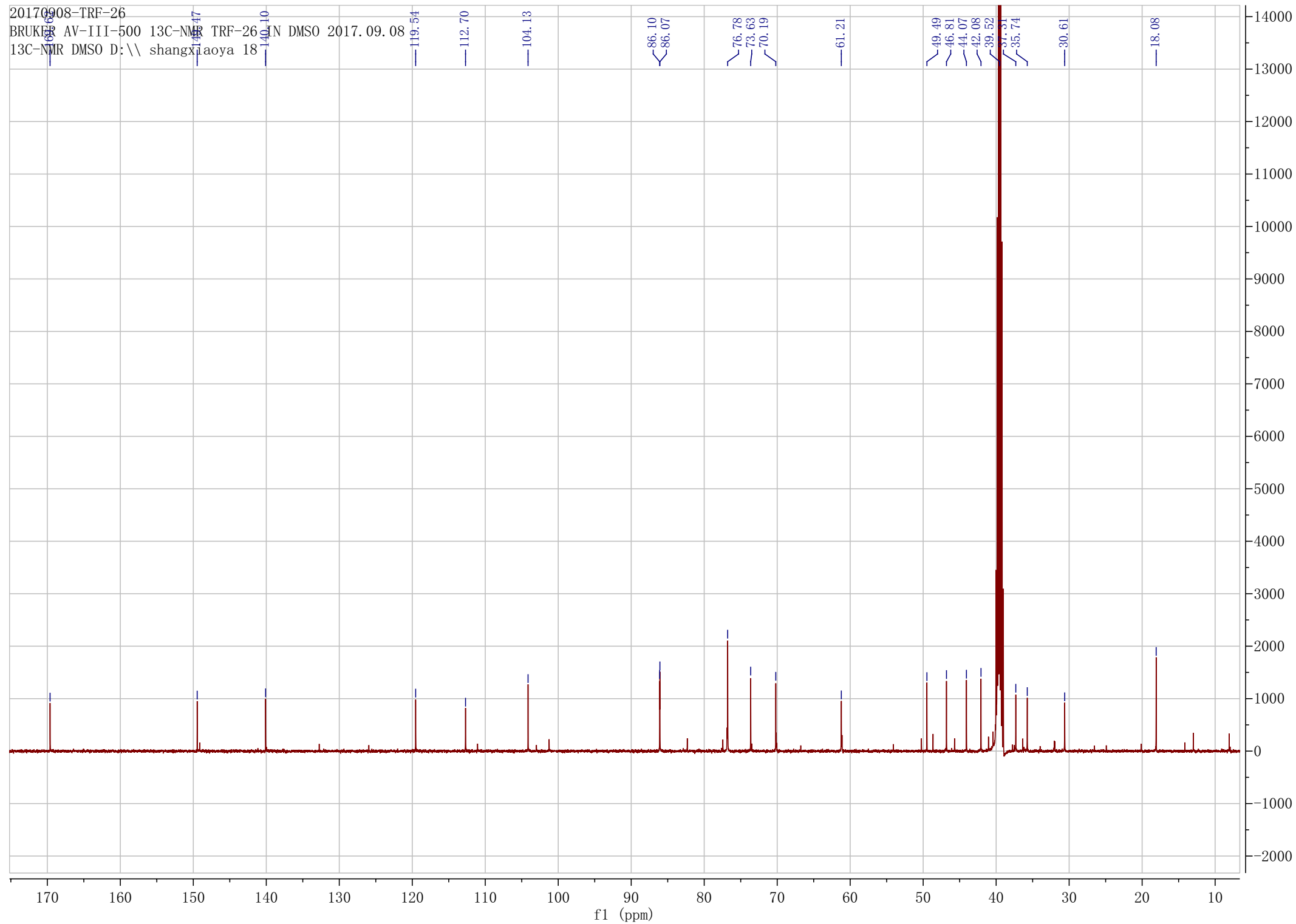

Supplement: Supplementary file 1 [file molecules-24-01701-s001.zip › molecules-489408-proofreading done-Suppl/data of compounds 1-10/Compound 4-C.pdf]

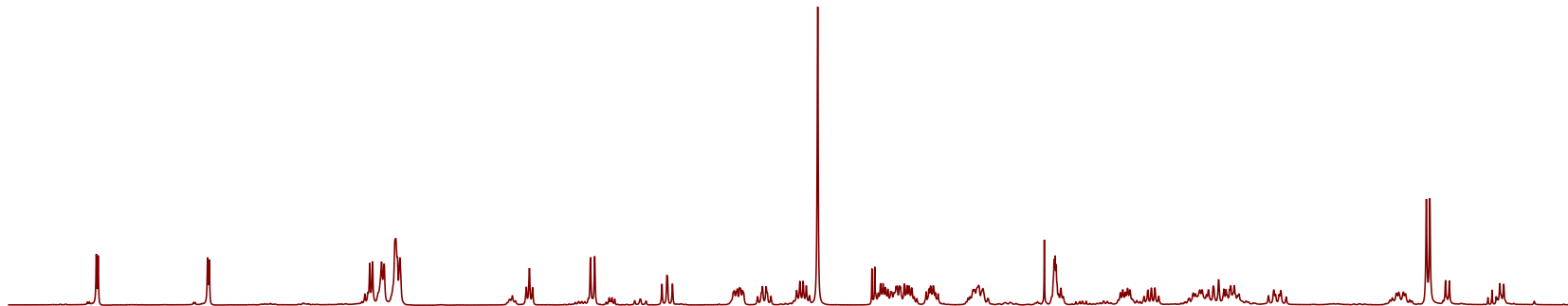

20171101-TRF-26  
BRUKER AV-III-500 COSY-NMR TRF-26 IN DMSO 2017.11.01  
COSYGPMFSW DMSO D:\\ shangxiaoya 4

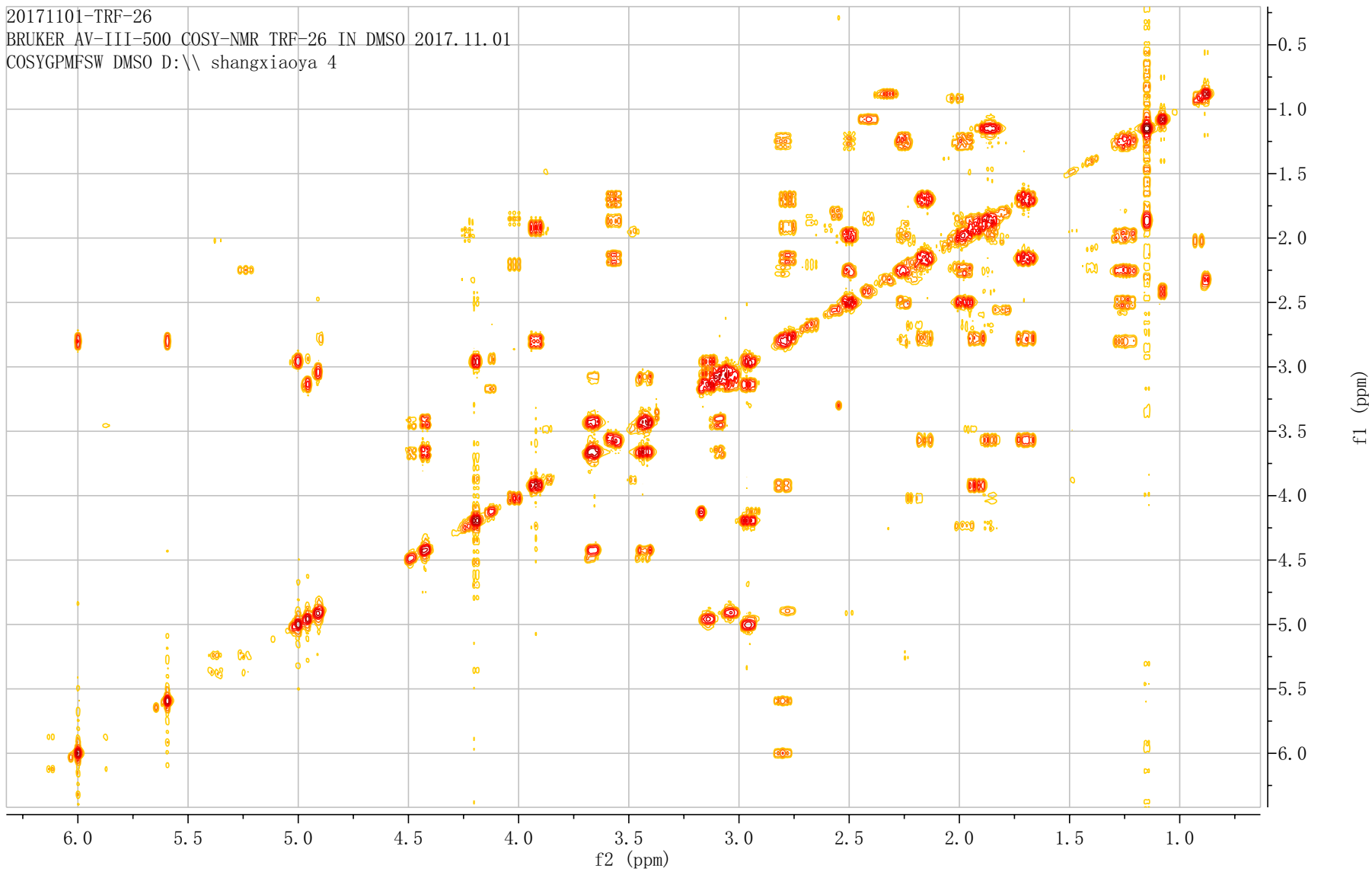

Supplement: Supplementary file 1 [file molecules-24-01701-s001.zip › molecules-489408-proofreading done-Suppl/data of compounds 1-10/Compound 4-COSY.pdf]

20170908-TRF-26  
BRUKER AV-500 1H-NMR TRF-26 IN DMSO-2017.09.08  
1H-NMR DMSO D:\\shangxiaoya-18

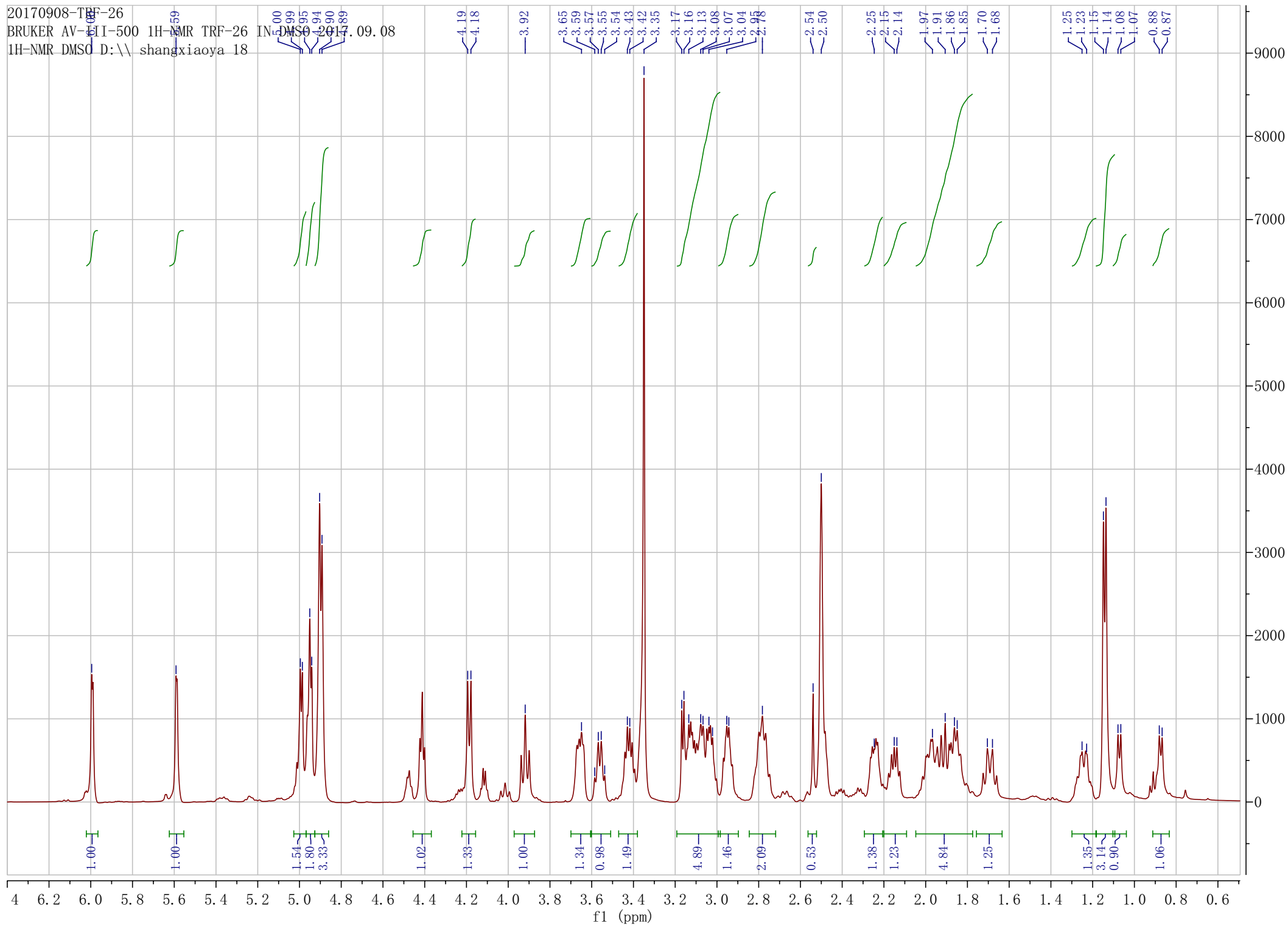

Supplement: Supplementary file 1 [file molecules-24-01701-s001.zip › molecules-489408-proofreading done-Suppl/data of compounds 1-10/Compound 4-H.pdf]

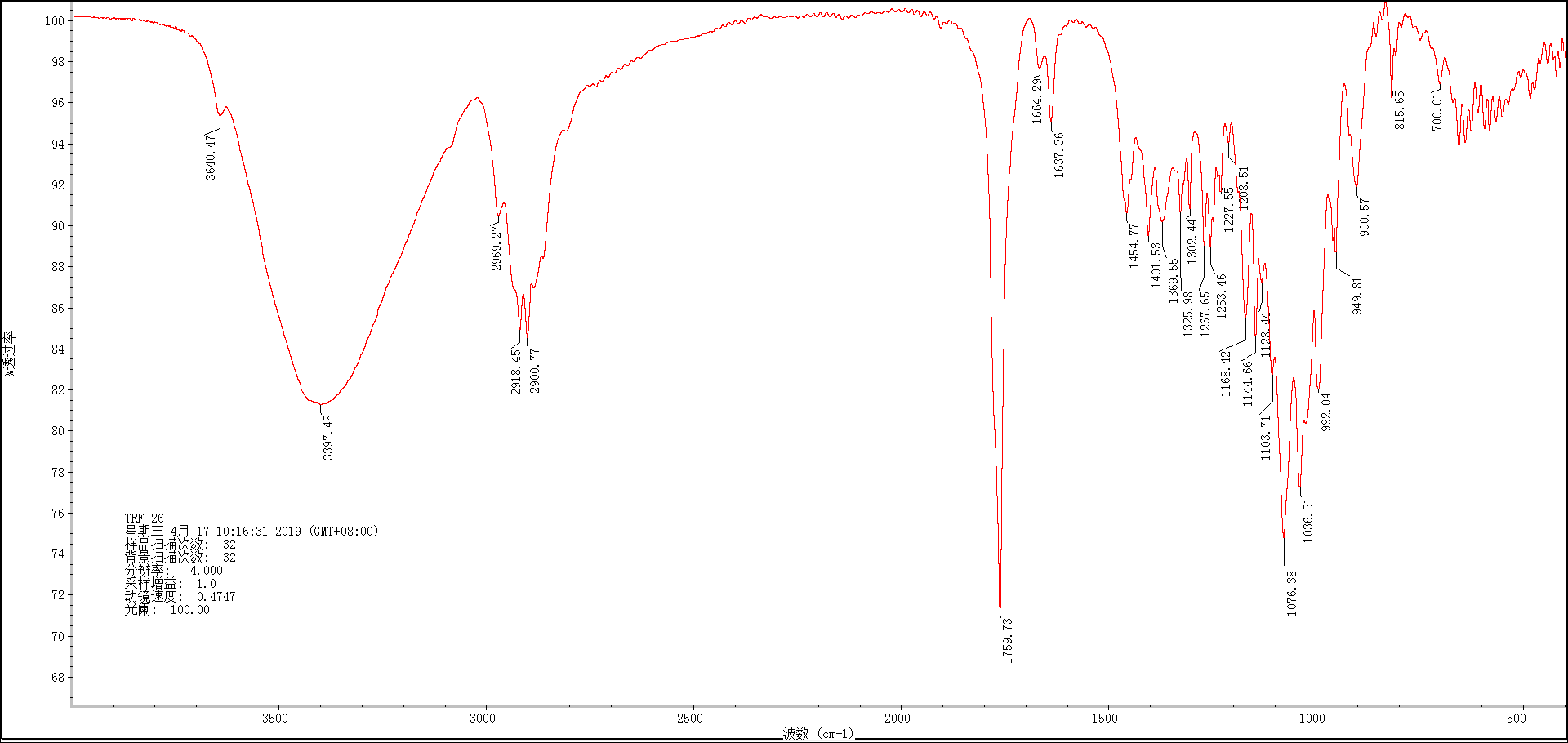

Supplement: Supplementary file 1 [file molecules-24-01701-s001.zip › molecules-489408-proofreading done-Suppl/data of compounds 1-10/Compound 4-IR.TIF]

TRF-26\_Pos\_FullMs #831 RT: 4.21 AV: 1 NL: 4.71E8  
T: FTMS + p ESI Full ms [50.0000-500.0000]

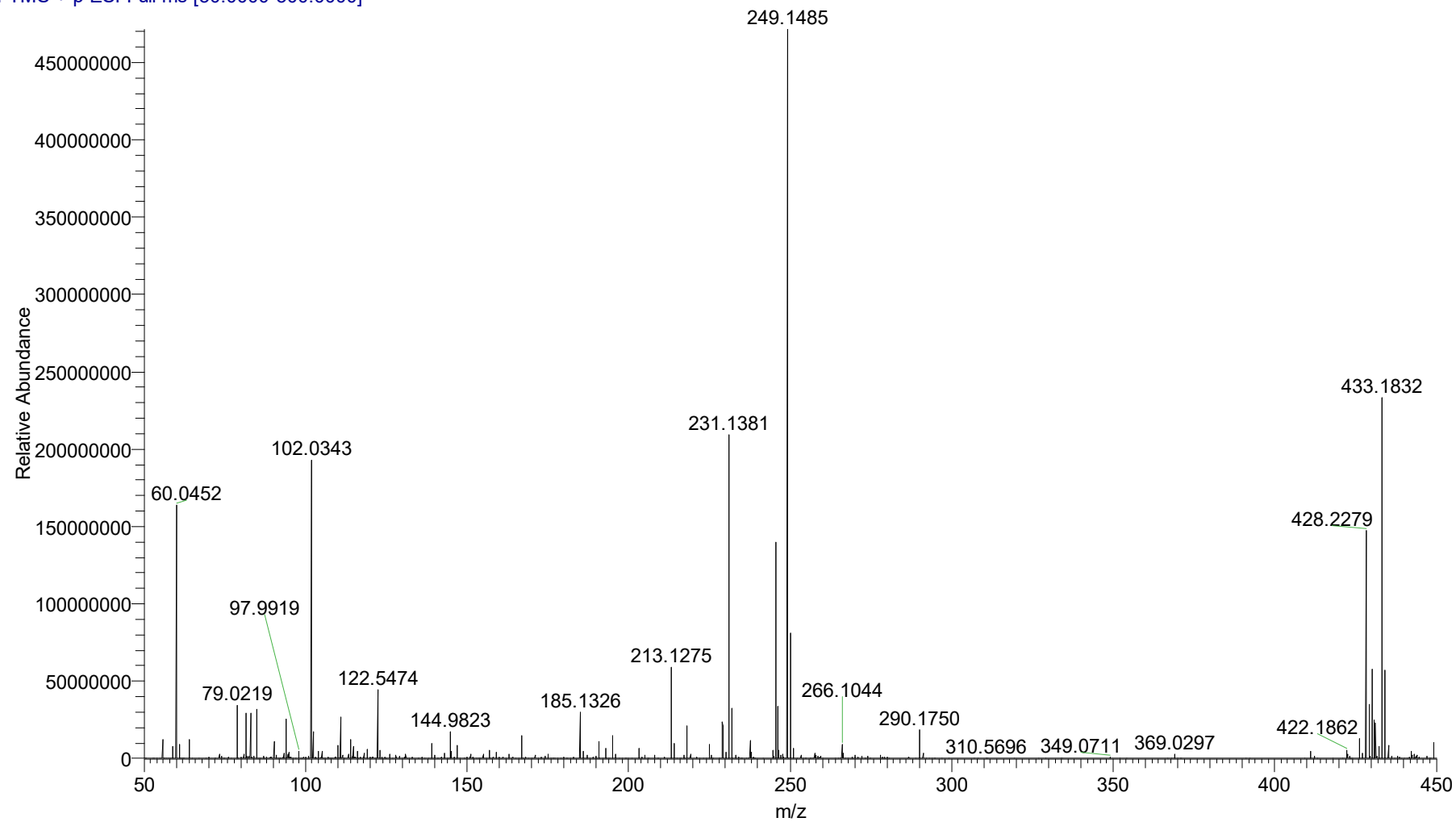

Supplement: Supplementary file 1 [file molecules-24-01701-s001.zip › molecules-489408-proofreading done-Suppl/data of compounds 1-10/Compound 4-MS.pdf]

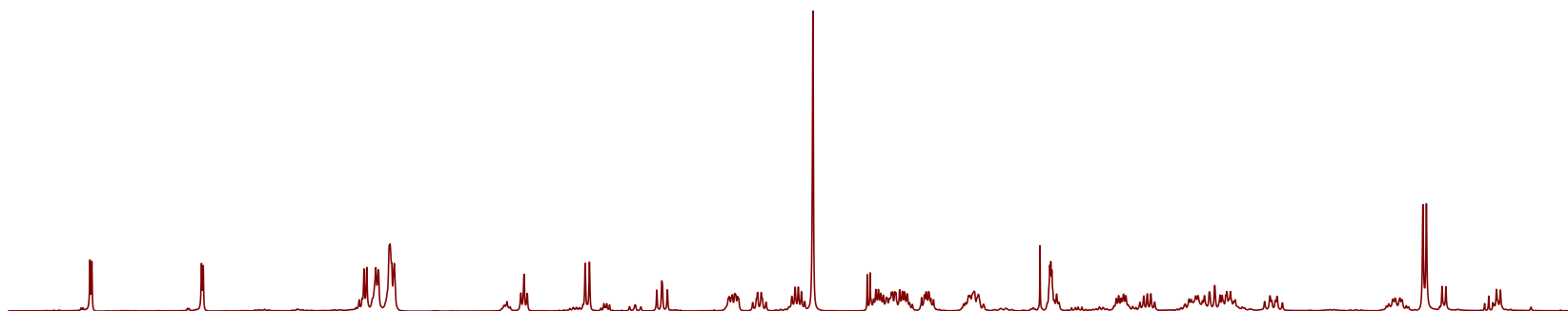

20171101-TRF-26  
BRUKER AV-III-500 COSY-NMR TRF-26 IN DMSO 2017.11.01  
HSQCETGPSI DMSO D:\\ shangxiaoya 4

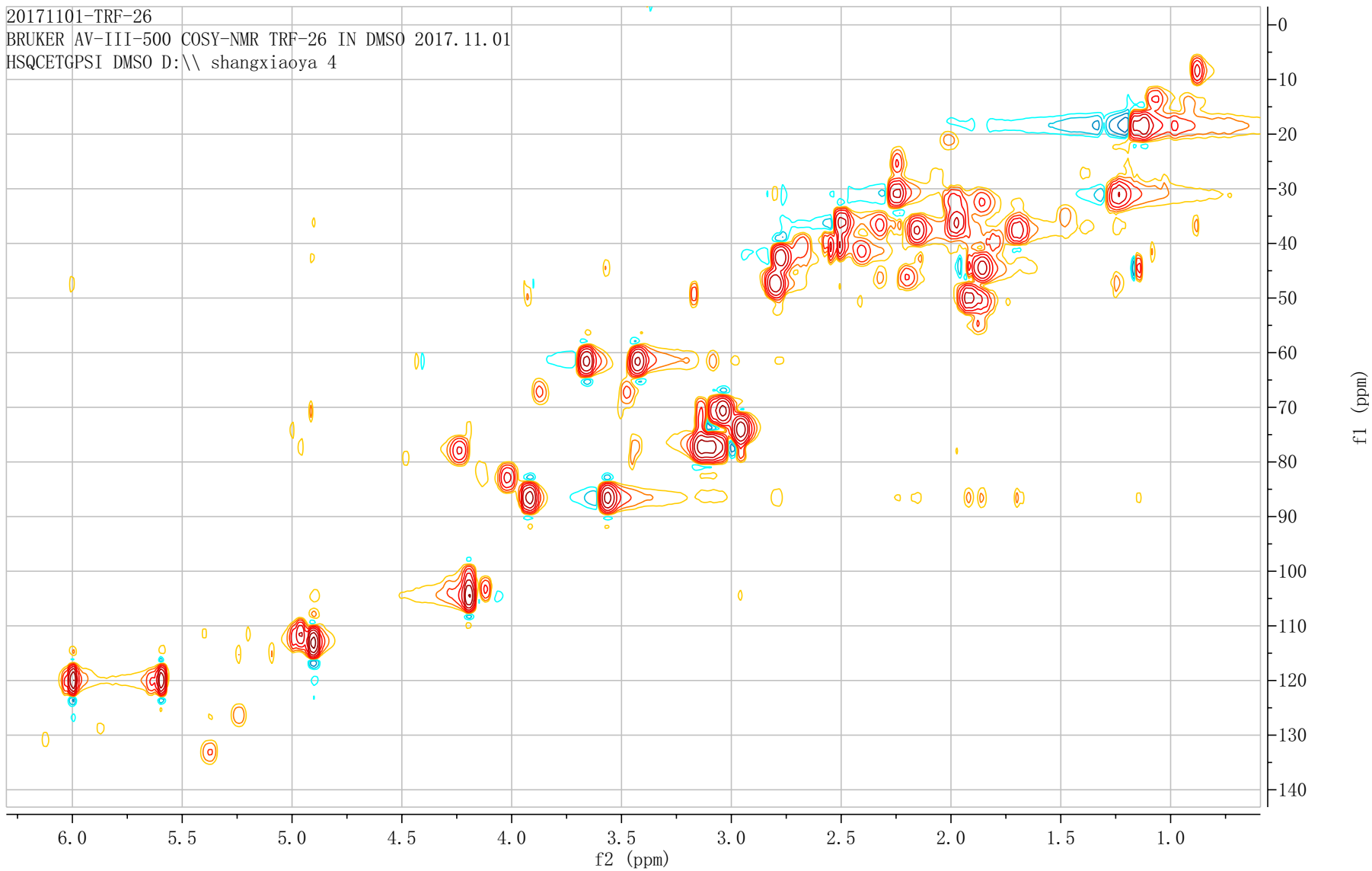

Supplement: Supplementary file 1 [file molecules-24-01701-s001.zip › molecules-489408-proofreading done-Suppl/data of compounds 1-10/Compound 4-QC.pdf]

20180429-TRF-41

<sup>13</sup>C-NMR MeOD D:\ shangxiaoya 34

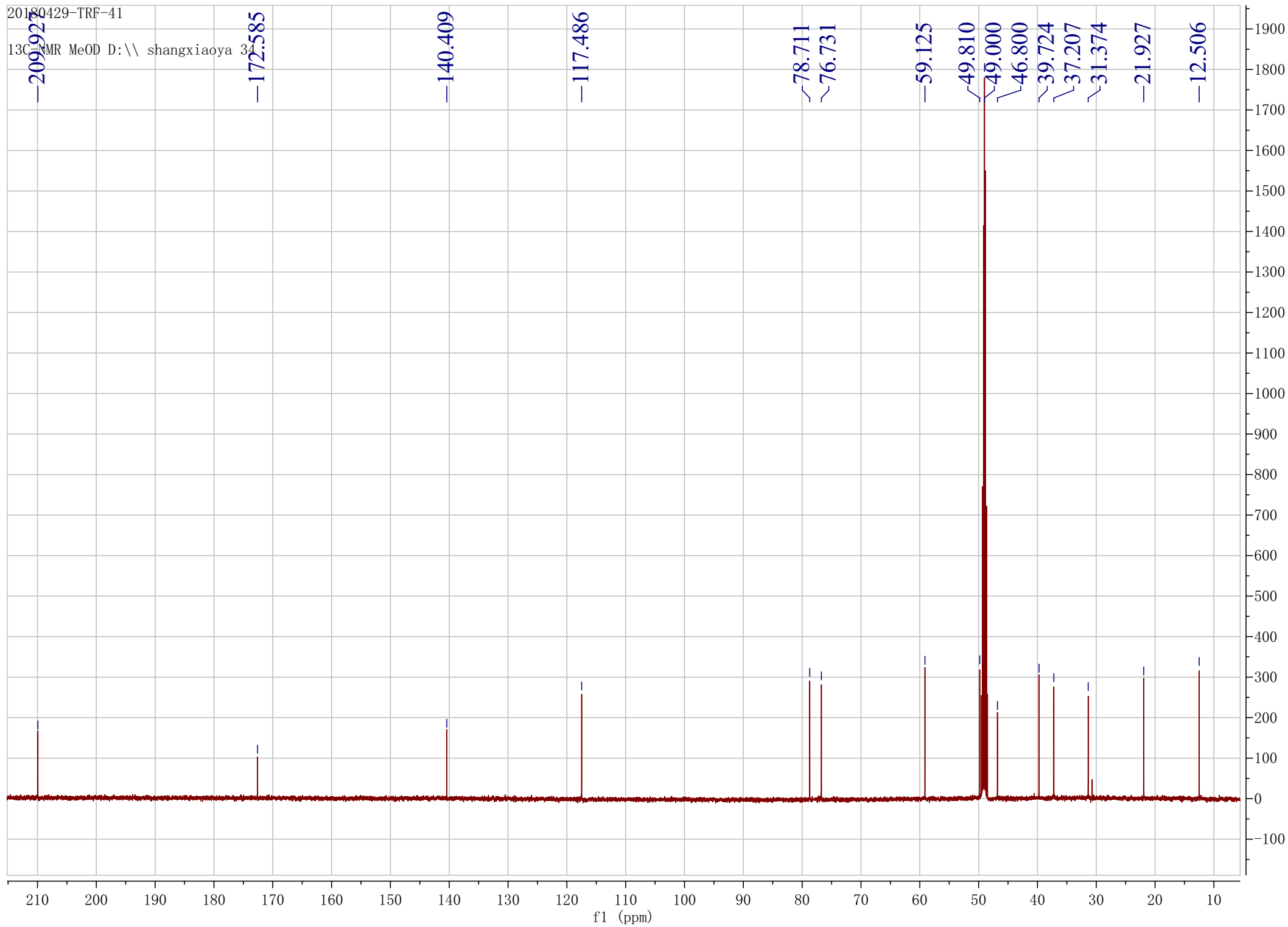

Supplement: Supplementary file 1 [file molecules-24-01701-s001.zip › molecules-489408-proofreading done-Suppl/data of compounds 1-10/Compound 5-C.pdf]

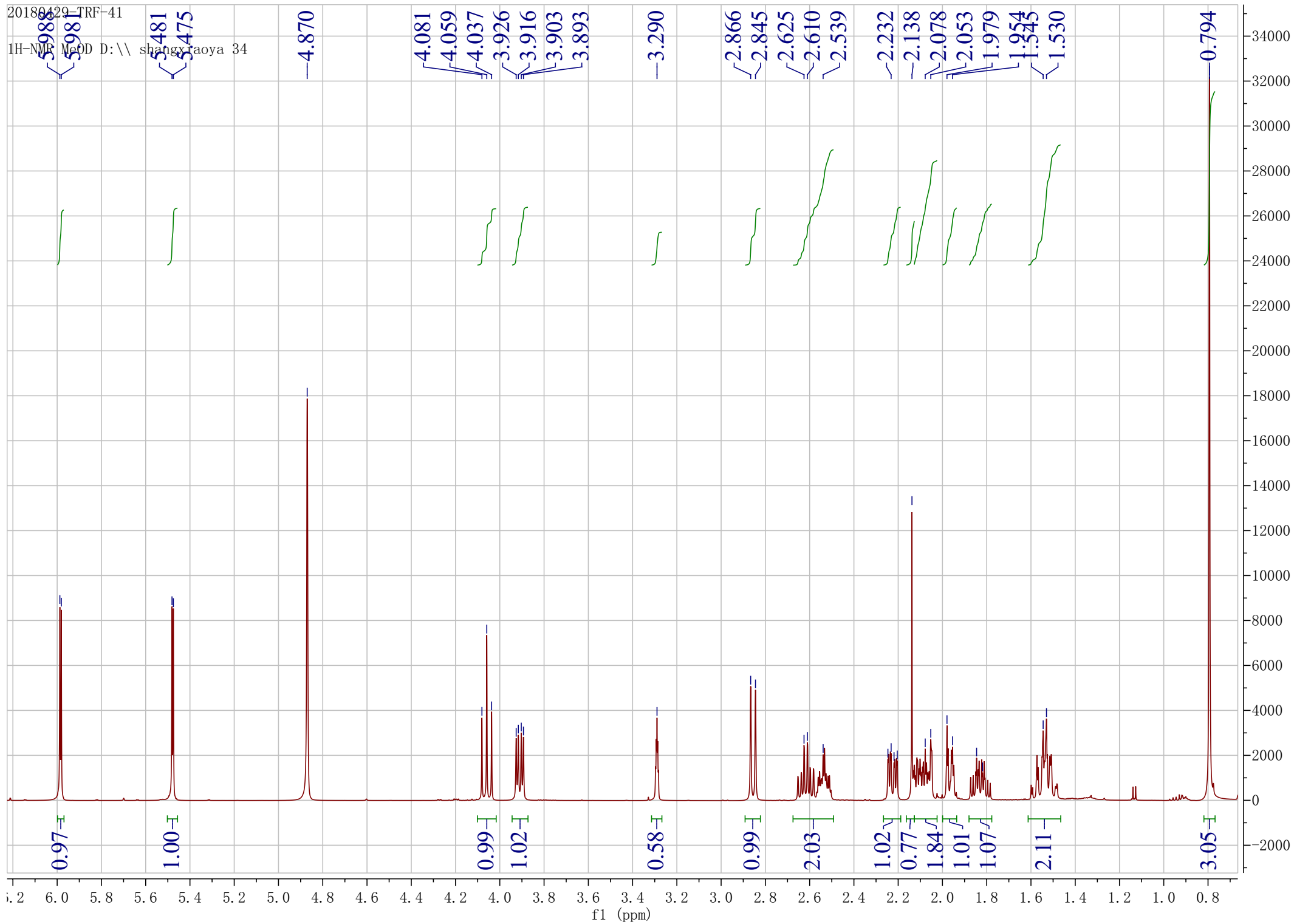

Supplement: Supplementary file 1 [file molecules-24-01701-s001.zip › molecules-489408-proofreading done-Suppl/data of compounds 1-10/Compound 5-H.pdf]

20180429-TRF-42

<sup>13</sup>C-NMR MeOD D:\shangxiangya-35

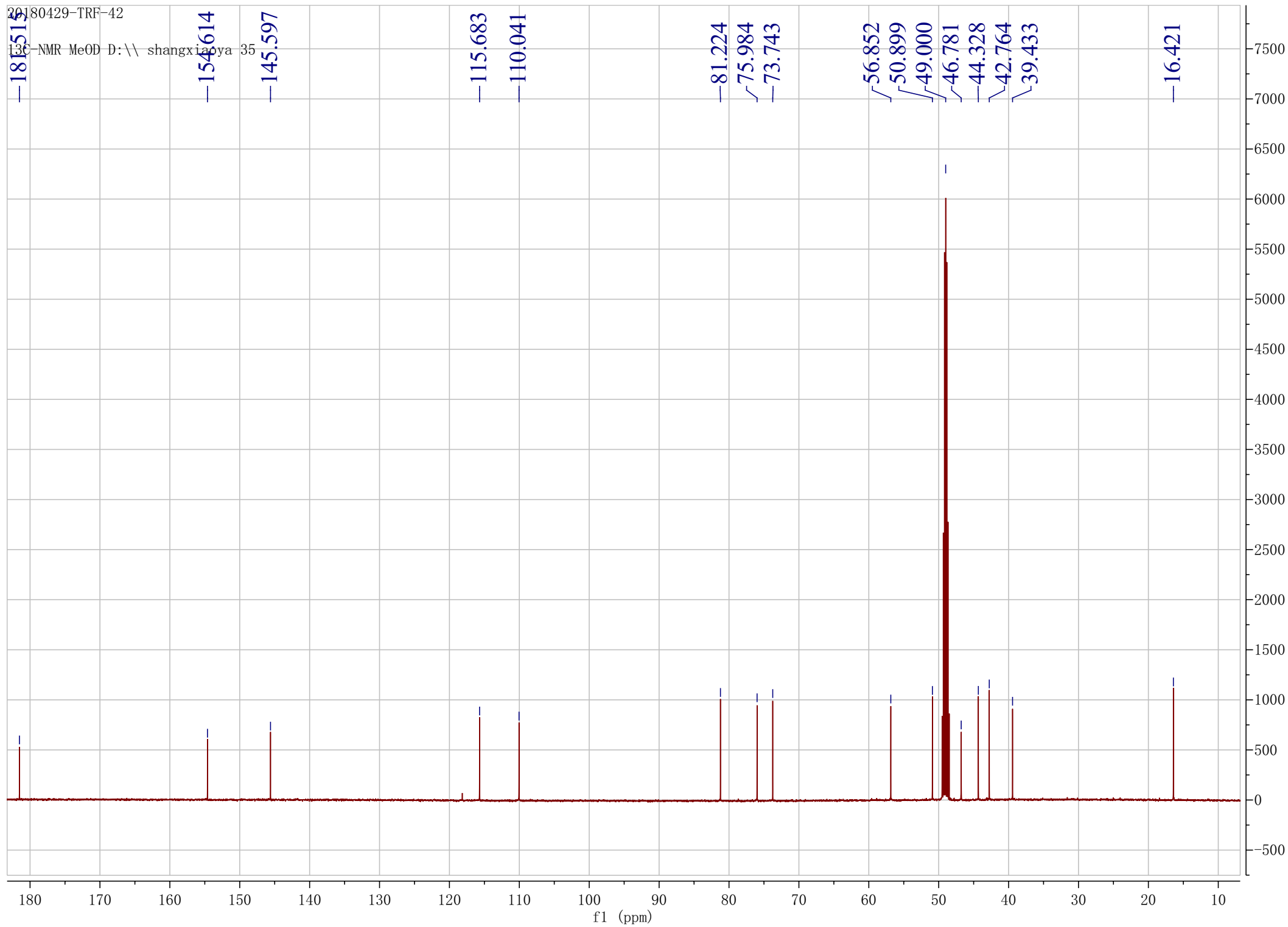

Supplement: Supplementary file 1 [file molecules-24-01701-s001.zip › molecules-489408-proofreading done-Suppl/data of compounds 1-10/Compound 6-C.pdf]

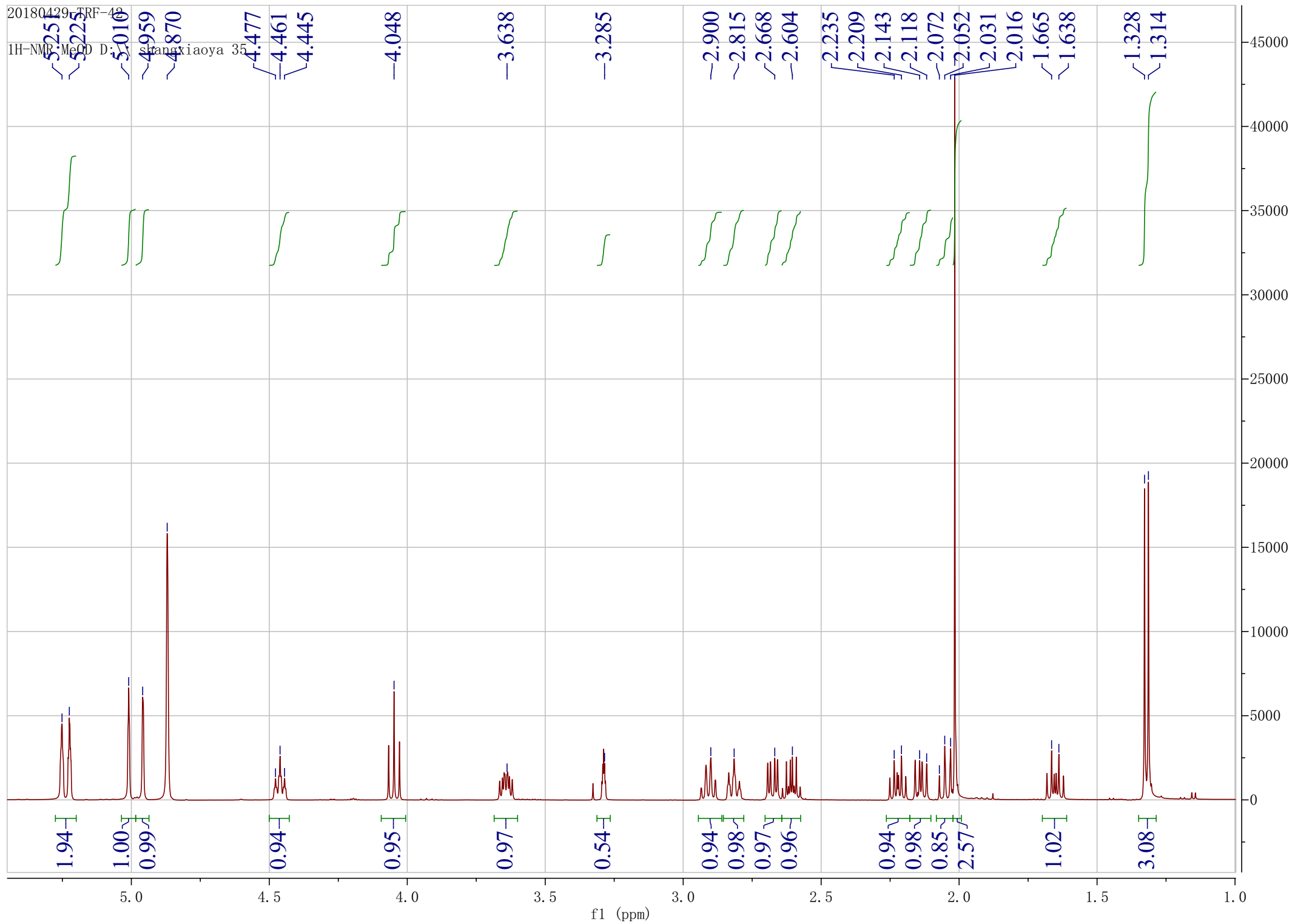

Supplement: Supplementary file 1 [file molecules-24-01701-s001.zip › molecules-489408-proofreading done-Suppl/data of compounds 1-10/Compound 6-H.pdf]

20180429-TRF-43

<sup>13</sup>C-NMR MeOD D:\ shangxiaoya 36

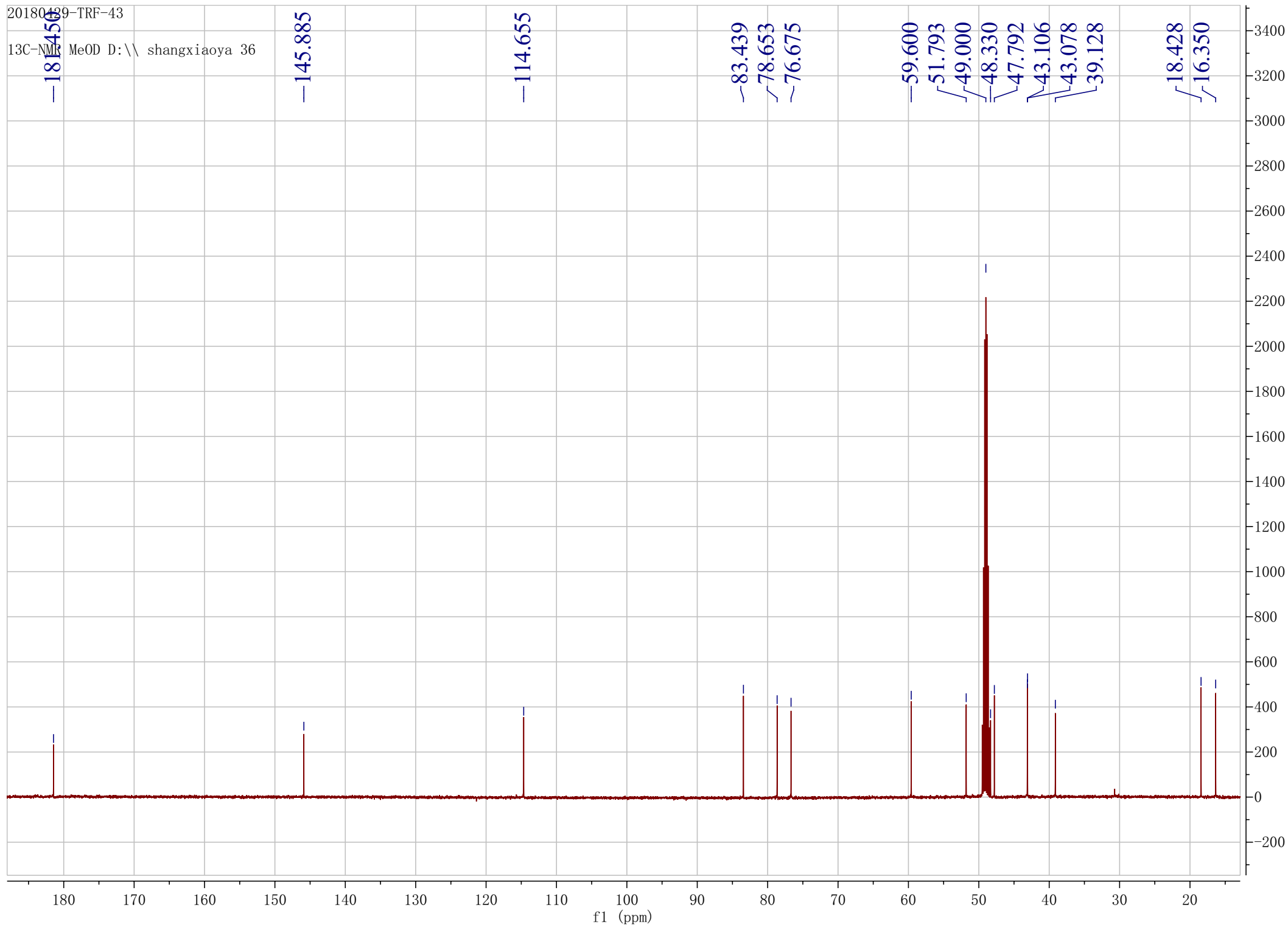

Supplement: Supplementary file 1 [file molecules-24-01701-s001.zip › molecules-489408-proofreading done-Suppl/data of compounds 1-10/Compound 7-C.pdf]

20180429-TRF\_43

1H-NMR: MeOD-d<sub>4</sub> \\ shangxiaoya 36

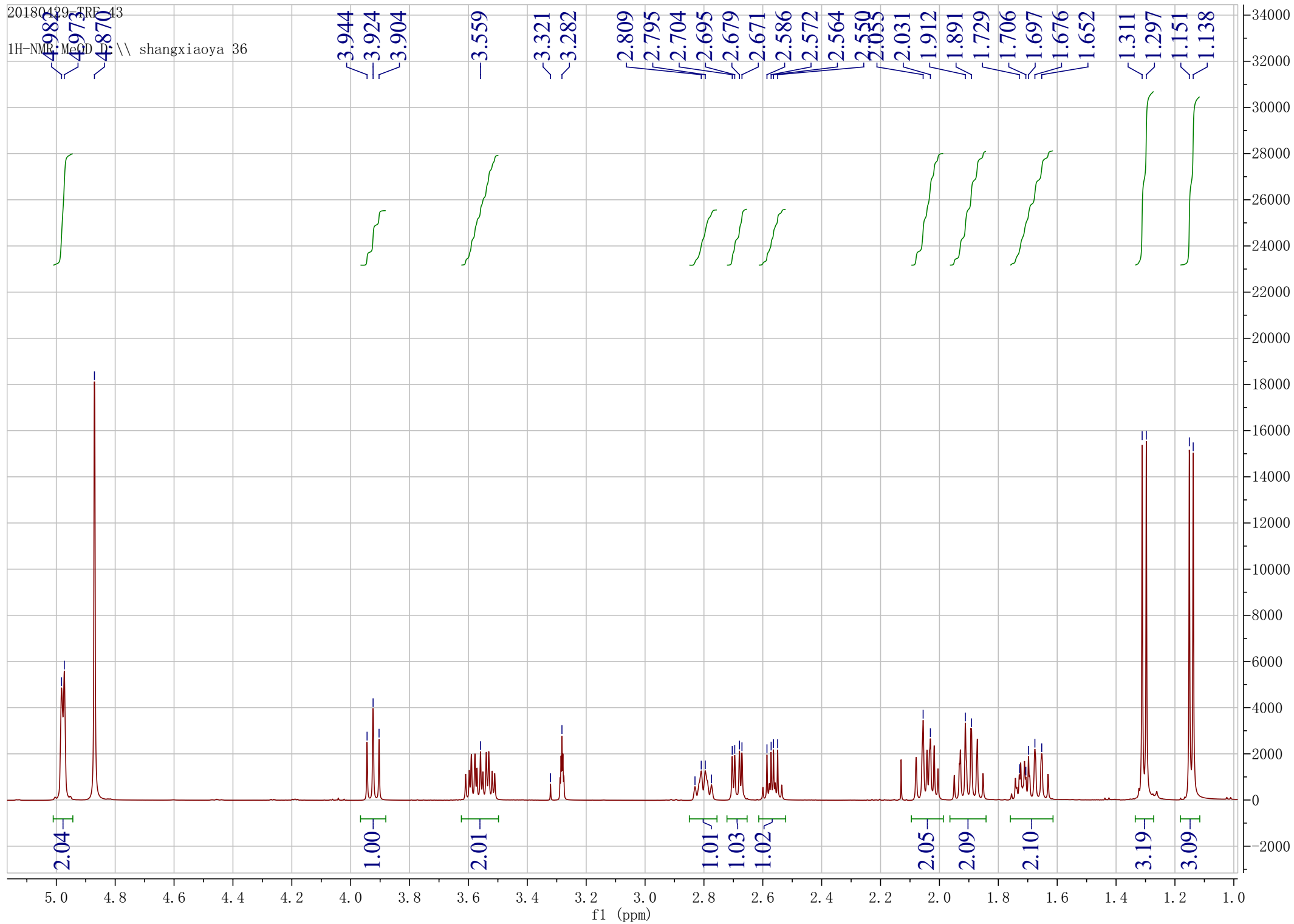

Supplement: Supplementary file 1 [file molecules-24-01701-s001.zip › molecules-489408-proofreading done-Suppl/data of compounds 1-10/Compound 7-H.pdf]

20170908-TRF-25  
BRUKER AVIII-500 13C-NMR TRF-25 IN DMSO 2017.09.08  
13C-NMR DMSO D:\shangxiaoya 17

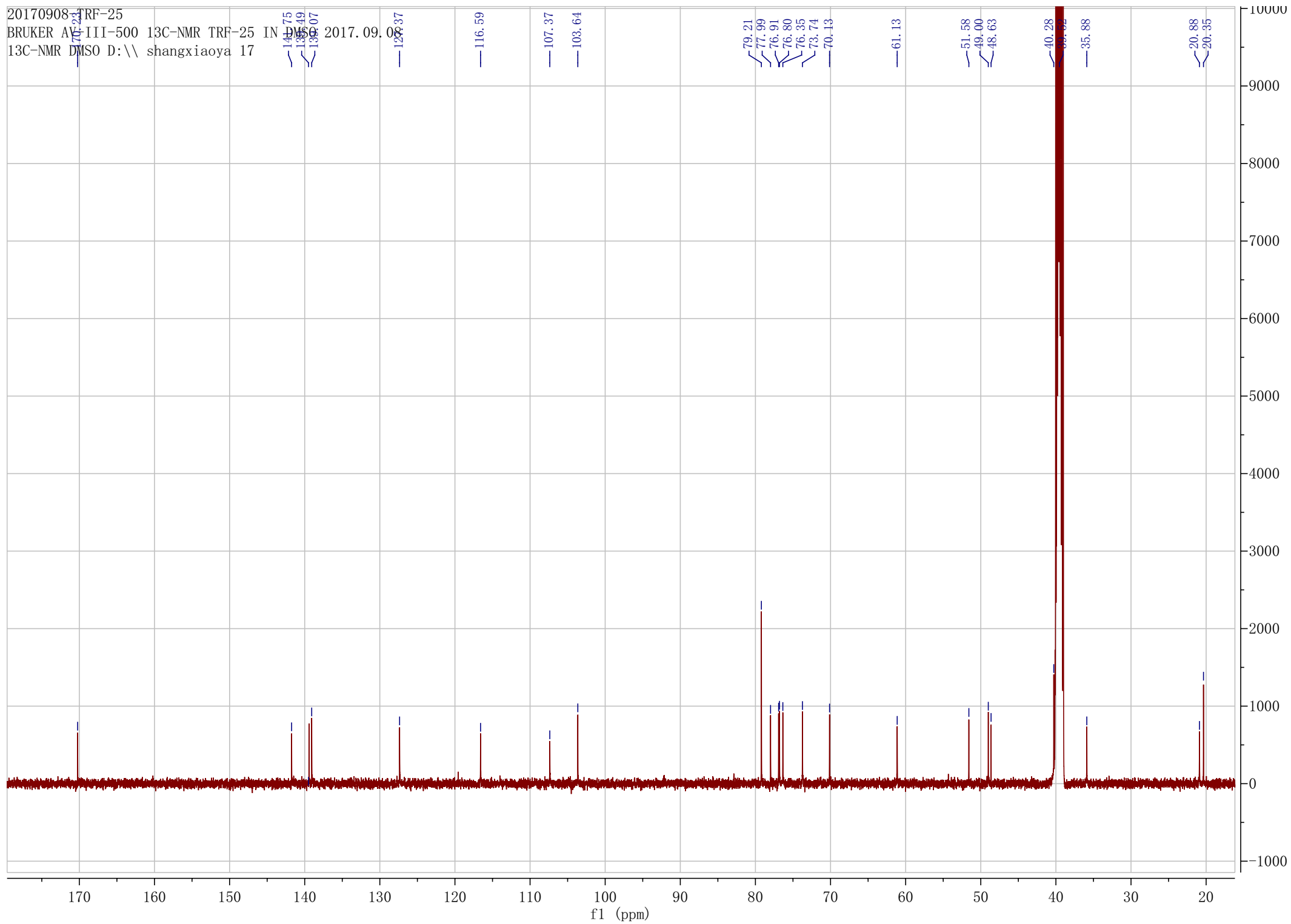

Supplement: Supplementary file 1 [file molecules-24-01701-s001.zip › molecules-489408-proofreading done-Suppl/data of compounds 1-10/Compound 8-C.pdf]

20170908-TRF-25  
BRUKER AVIII-500 1H-NMR TRF-25 IN DMSO 2017.09.08  
1H-NMR DMSO D:\shangxiaoya 17

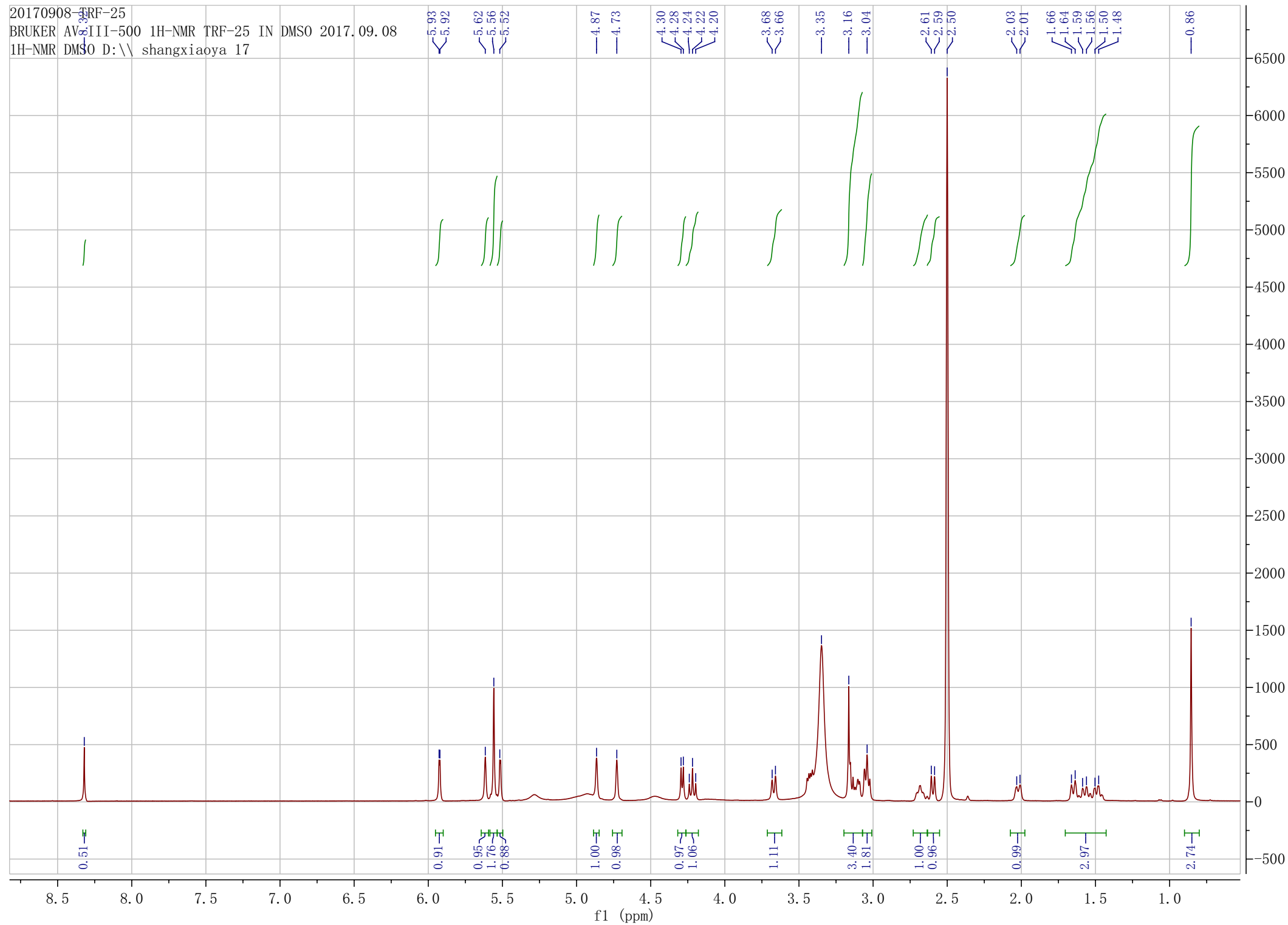

Supplement: Supplementary file 1 [file molecules-24-01701-s001.zip › molecules-489408-proofreading done-Suppl/data of compounds 1-10/Compound 8-H.pdf]

20170908-TRF-27  
BRUKER AV-III-500 13C-NMR TRF-27 IN DMSO 2017.09.08  
13C-NMR DMSO D:\\shangxiaoya\\19

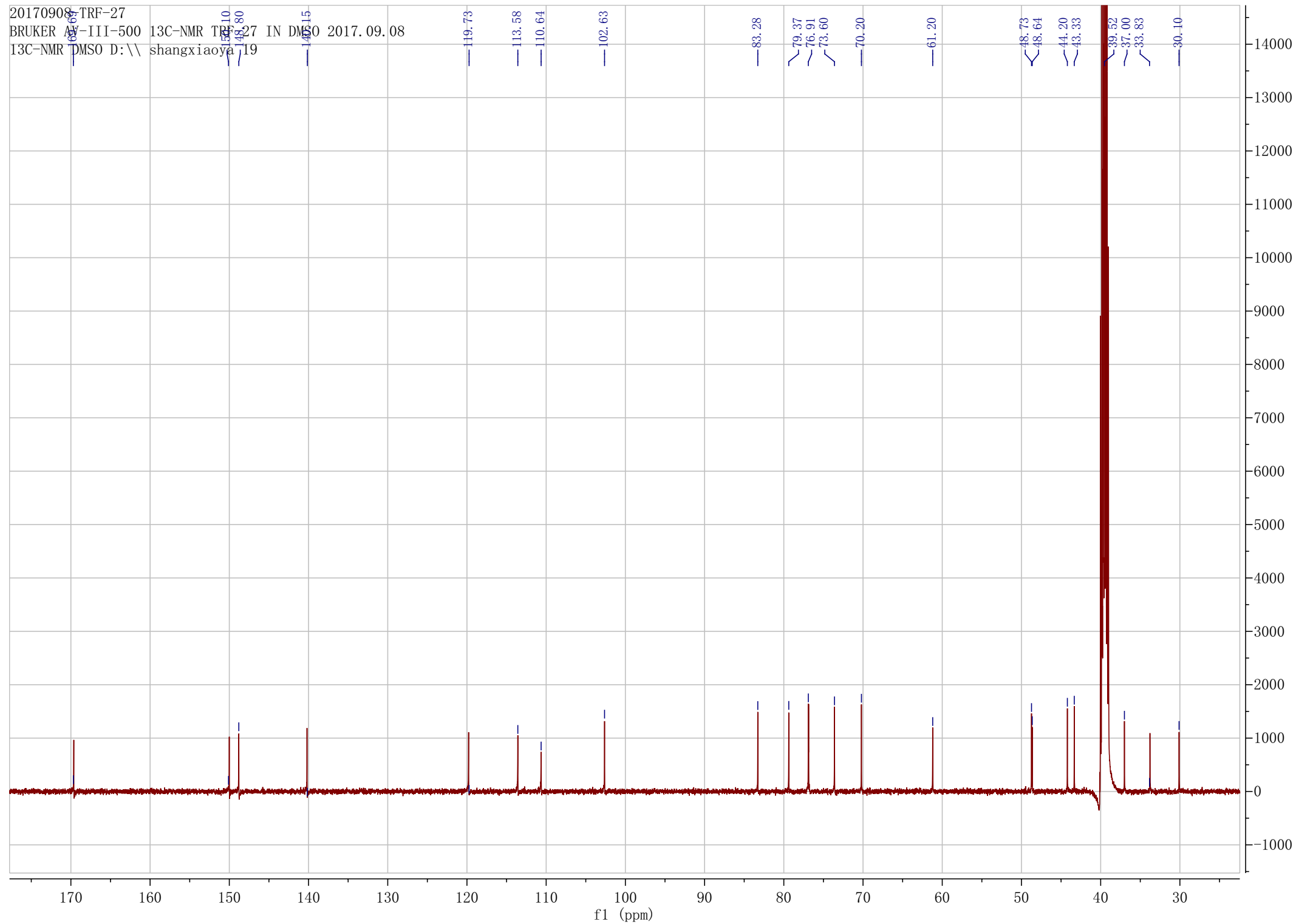

Supplement: Supplementary file 1 [file molecules-24-01701-s001.zip › molecules-489408-proofreading done-Suppl/data of compounds 1-10/Compound 9-C.pdf]

20170908-TRF-27  
BRUKER AV-III-500 1H-NMR TRF-27 IN DMSO 2017.09.08  
1H-NMR DMSO D:\shangxiaoya 19

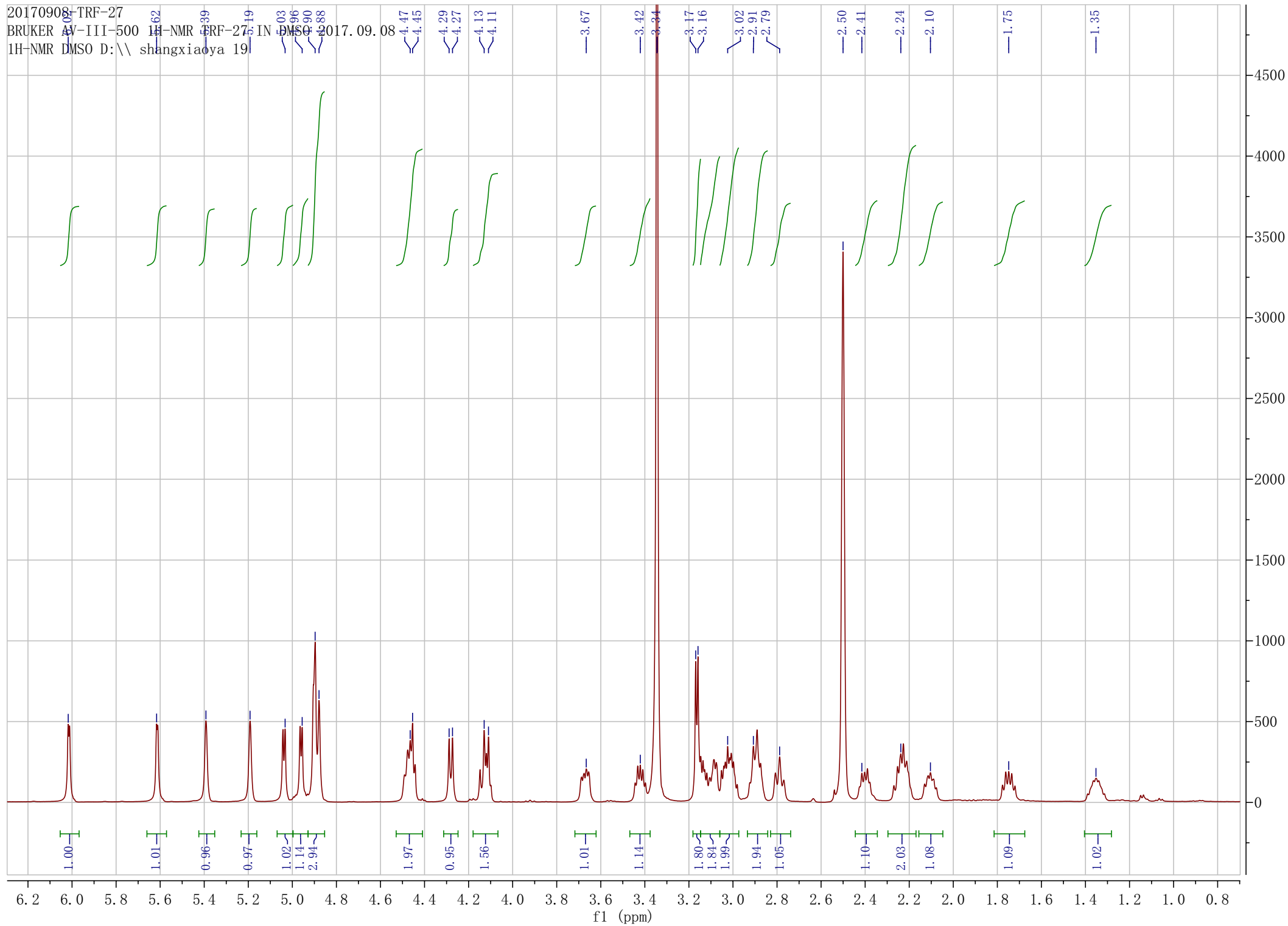

Supplement: Supplementary file 1 [file molecules-24-01701-s001.zip › molecules-489408-proofreading done-Suppl/data of compounds 1-10/Compound 9-H.pdf]

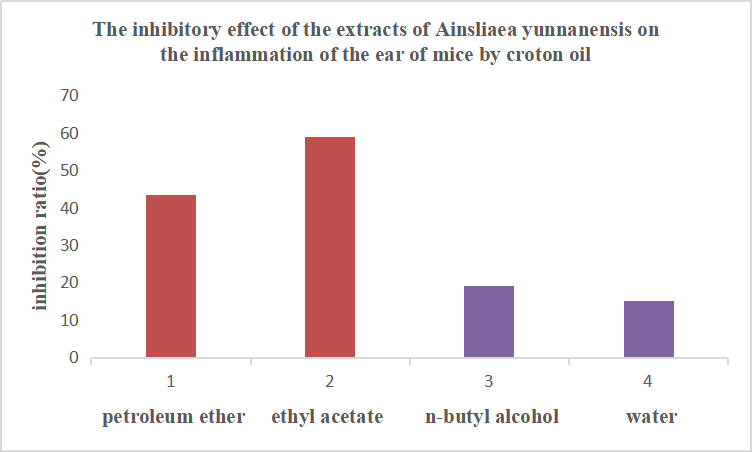

Supplement: Supplementary file 1 [file molecules-24-01701-s001.zip › molecules-489408-proofreading done-Suppl/Figure S1.png]
